# The Genome Sequence of the North-European Cucumber (*Cucumis sativus* L.) Unravels Evolutionary Adaptation Mechanisms in Plants

**DOI:** 10.1371/journal.pone.0022728

**Published:** 2011-07-28

**Authors:** Rafał Wóycicki, Justyna Witkowicz, Piotr Gawroński, Joanna Dąbrowska, Alexandre Lomsadze, Magdalena Pawełkowicz, Ewa Siedlecka, Kohei Yagi, Wojciech Pląder, Anna Seroczyńska, Mieczysław Śmiech, Wojciech Gutman, Katarzyna Niemirowicz-Szczytt, Grzegorz Bartoszewski, Norikazu Tagashira, Yoshikazu Hoshi, Mark Borodovsky, Stanisław Karpiński, Stefan Malepszy, Zbigniew Przybecki

**Affiliations:** 1 Department of Plant Genetics, Breeding and Biotechnology, Faculty of Horticulture and Landscape Architecture, Warsaw University of Life Sciences - SGGW, Nowoursynowska, Warsaw, Poland; 2 Center for Bioinformatics and Computational Genomics, Joint Wallace H. Coulter Georgia Tech and Emory Department of Biomedical Engineering, School of Computational Science and Engineering, Georgia Institute of Technology, Atlanta, Georgia, United States of America; 3 Department of Living Design and Information Science, Faculty of Human Development, Hiroshima Jogakuin University, Higashi-ku, Japan; 4 Department of Plant Science, Tokai University, Minamiaso-mura, Kumamoto, Japan; Instituto de Biología Molecular y Celular de Plantas, Spain

## Abstract

Cucumber (*Cucumis sativus* L.), a widely cultivated crop, has originated from Eastern Himalayas and secondary domestication regions includes highly divergent climate conditions e.g. temperate and subtropical. We wanted to uncover adaptive genome differences between the cucumber cultivars and what sort of evolutionary molecular mechanisms regulate genetic adaptation of plants to different ecosystems and organism biodiversity. Here we present the draft genome sequence of the *Cucumis sativus* genome of the North-European Borszczagowski cultivar (line B10) and comparative genomics studies with the known genomes of: *C. sativus* (Chinese cultivar – Chinese Long (line 9930)), *Arabidopsis thaliana*, *Populus trichocarpa* and *Oryza sativa*. Cucumber genomes show extensive chromosomal rearrangements, distinct differences in quantity of the particular genes (e.g. involved in photosynthesis, respiration, sugar metabolism, chlorophyll degradation, regulation of gene expression, photooxidative stress tolerance, higher non-optimal temperatures tolerance and ammonium ion assimilation) as well as in distributions of abscisic acid-, dehydration- and ethylene-responsive *cis*-regulatory elements (CREs) in promoters of orthologous group of genes, which lead to the specific adaptation features. Abscisic acid treatment of non-acclimated *Arabidopsis* and *C. sativus* seedlings induced moderate freezing tolerance in *Arabidopsis* but not in *C. sativus*. This experiment together with analysis of abscisic acid-specific CRE distributions give a clue why *C. sativus* is much more susceptible to moderate freezing stresses than *A. thaliana*. Comparative analysis of all the five genomes showed that, each species and/or cultivars has a specific profile of CRE content in promoters of orthologous genes. Our results constitute the substantial and original resource for the basic and applied research on environmental adaptations of plants, which could facilitate creation of new crops with improved growth and yield in divergent conditions.

## Introduction

The cultivated cucumber (*Cucumis sativus* L.), an important species for the global food market (http://faostat.fao.org), represents a model organism for investigations of organellar genetics [1]–[3], flower sex determination [4], mechanisms of somatic embryogenesis [5]–[7] epigenetics of various stresses in tissue culture [8], [9] and various aspects of chloroplast gene regulation (e.g. editing, tilling macroarray) [10], [11]. Genetic and cultivation research of that species is conducted by many international groups including the International Cucurbit Genomics Initiative (ICuGI) – (http://www.icugi.org/cgi-bin/ICuGI/misc/project.cgi); and the USDA-ARS at the University of Wisconsin, Madison. Cucumber is cultivated in habitats ranging from the Northern regions of the temperate climate zone to the tropics. Spatial isolation and the need to adapt to diverse environmental conditions have induced a requirement for various adaptation mechanisms based upon polymorphisms within the 367 Mbp cucumber genome [12]. These factors have inspired us to undertake the *de novo* sequencing of genome of the Northern European cucumber. While this work was in progress the genomic sequence of the Chinese Long cultivar of cucumber which is known as line 9930 became available [13]. This has made original comparative studies between the two cultivars possible.

The evolution of organisms is driven by their need to reproduce as efficiently as possible in a given habituate. The optimization of that Darwinian fitness is known to include mechanisms of, for example, point mutations and chromosomal rearrangements leading to changes in gene quality and quantity as well as *cis*-regulatory element shuffling, which facilitate formation of new species and varieties. The speed of evolution is dependent on the stringency of abiotic and biotic stresses and on the organism ability to respond to the given stringency. Highly divergent environmental conditions of species domestication, which are different from its place of origin, speed up this process. It is known that, abiotic stresses caused by starvation, high or low temperatures, UV radiation or treatment with hydrogen peroxide cause chromosomal rearrangements in the form of translocations, deletions, inversions, amplifications and chromosome fusions, as well as aneuploidy. These chromosomal rearrangements are stabilized over the generations and through changes in gene content lead to acquisition of resistance, as it was shown in e.g. yeast and plants [14]–[20]. Changes in regulation of gene expression due to regulatory networks rewiring could constitute other and faster mechanism of adaptations. Plants and animals have homologous and analogous regulatory mechanisms of stress responses, which include the selective activation/deactivation of appropriate trans-regulatory factors (TRFs) that specifically interact with *cis*-regulatory elements (CREs), in order to induce or suppress gene expression. This leads to the establishment of a new optimal adaptation status [21]–[24].

CREs are short DNA sequences generally composed of several nucleotides in the promoter region of a gene, for which TRFs have specific affinity. CREs and TRFs provide the molecular basis for transcriptional regulation both in plants and in animals. Induction or suppression of genes expression may depend upon the presence of single CRE or combinations of CREs as well as the orientation of CREs with respect to the gene [25], [26]. The best characterized CREs in plants include abscisic acid responsive element (ABRE (sequence – ACGTGTC)) which, as implied by its name, is linked to the response to abscisic acid (ABA), dehydration responsive element (DRE (also known as CRT), sequence - A/GCCGAC), an element induced by drought, salinity and frost, and ethylene responsive element (ERE (also known as GCC-box), sequence – AGCCGCC), an element linked to the response to ethylene. Abscisic acid is a hormone which plays an important role in the integrated response of plants to stresses caused by drought, cold, excessive light, pathogen attack and salinity [27], [28]. Ethylene plays an important role in regulation of many aspects of plant life including flowering, fruit ripening, root hypoxia responses, and other programmed cell death-dependent processes [21], [29].

Here we present the draft genome sequence of northern European cucumber cultivar Borszczagowski line B10 assembled by a hybrid method, structural and functional annotations and its comparisons with the highly divergent [30] Asian cucumber cultivar Chinese Long line 9930 [13] and the genomes of three other species (*Arabidopsis thaliana*, *Populus trichocarpa*, *Oryza sativa*). Shotgun sequencing was performed using the 454 Titanium technique at 12× genome coverage (8× genome coverage with single reads and 4× genome coverage with paired reads (3 Kbp insert size fragments). Additionally, the ends of inserts of a genomic BAC library were sequenced using the Sanger method. We analyzed SNP/INDEL polymorphism, genome rearrangements, compared functional groups of genes between the two cucumbers and also performed comparative analysis of the above CREs content for all the above five species.

Presented here comparative genomic studies of five plant genomes provide a strong support for the previous suggestions [31], [32] that optimization of the Darwinian fitness of a given species or variety is determined by the evolution of the content of the genome (expressed as changes in number and quality of genes) and mutations in some CRE. However, our results suggests that species- or even cultivar-specific evolution of ABRE, DRE and ERE regulons, due to extensive CRE shuffling is faster than the evolution of the gene introns and exons. Such comparative genomic studies that also include CRE content analysis in groups of orthologous gene promoters supported by experiments will help in finding of new biotechnological solutions for a specific improvement of crops performance.

## Results

### Sequencing of the cucumber genome

#### Bacterial artificial chromosome (BAC) library

The number of clones of long genomic cucumber fragments derived from two libraries (*Mbo*I/*BamH*I and *Hind*III) was 33,792. The average length of the library inserts was 130 Kbp and the coverage of the genome with library BAC clones was 11.97×.

The basic characteristics of the BAC genomic library of line B10, such as average insert length and genome coverage, are comparable with the upper values of libraries previously described for plant species with similar genome sizes [33]–[36]. Three other cucumber BAC libraries contained shorter inserts with an average length of 105 Kbp in line S94 [37], 107 Kbp and 100.8 Kbp in the Suiseifushinari 2-go variety [38] and also had lower (2–2.5 fold) coverage of the genome and a smaller number of clones (10,000 to 15,000).

#### BAC end sequences (BESs)

Among the 33,792 inserts of the BAC library obtained after sequencing with the Sanger method, 66,776 sequences of clone ends were obtained (98.80% of the number of ends) (Table S1). Analysis of these sequences indicated the presence of 64,610 (96.76%) good quality fragments, without foreign DNA contamination. The percentage of chloroplast and mitochondrial genome sequences was 3.24%, and 0.46%, respectively. The remaining 62,217 (93.17%) ends of library clones were defined as sequences of nuclear origin (accession numbers: FI132140–FI136208, GS765762–GS766880, GS815969–GS874855). The average read length was 737 bp, and the total number of nucleotides in sequences of nuclear BESs was 45,853,929 bp, which corresponds to 12.49% of the genome. The average distance between sequences of BAC ends, which constitute unique genomic markers, was found to be 5500 bp. The participation of the obtained BAC end sequences and specifically of the paired ones was greater than 90% in each case and respectively 11% and 8% greater than the percentages described in previous publications [39], [40]. The number of organelle sequences present in the BESs was found to be similar to the numbers of sequences described in previous reports [41]–[43]. Two different libraries of the cucumber variety Suiseifushinari 2-go [38] contained 0.47% chloroplast sequences in *BamH*I clones and 0.26% chloroplast sequences in *EcoR*I clones. These libraries also had 0.40% mitochondrial sequences in *BamH*I clones and 0.20% mitochondrial sequences in *EcoR*I clones.

#### Sequence reads after pyrosequencing

The total number of fragments after paired (PA) and unpaired (UN) library ends sequencing was over 15 million with a total length of almost 4.5 billion bp. This represents 12× coverage of the genome. Purified sequences had an average length of 290 bp. The longest sequences (374 bp) were from the library of unpaired fragments and the shortest sequences (171 bp) were from the library of paired ends. Lengths and statistics of reads after pyrosequencing are presented in Figures S1A and S1B and Table S2. The reads obtained after sequencing the library of paired fragments were divided into sequences which were treated as: (a) unpaired because of: the absence of a linker, the occurrence of only a part of it, or the presence of many linker sequences, as well as (b) paired reads which gave the lowest coverage (1.50×).

The coverage generated by sequence reads was the lowest of those used so far in *de novo* sequencing of higher organisms using next generation sequencing technologies [44]–[46], but similar to 15× coverage recommended for as the minimal by 454 Life Sciences (Bradford, CT, USA) (http://454.com/downloads/DeNovoComplexGenomes_Flyer.pdf). A similar or lower coverage has only been used in sequencing of transcriptomes [47], [48], selected BAC clones [49], [50] and when using pyrosequencing as a supplement in capillary sequencing [51]. The average lengths of unpaired reads were close to those obtained during sequencing of the rice genome [49]. In turn, the paired Titanium reads were approximately two times longer than those described for GS FLX paired end libraries [45], [52]. The share of paired fragments was similar to the share described in the earlier reports [45].

#### Genome assembly

The results of both versions of genome assembly are presented in Table 1 and Table S3. In Table S4, the characteristics are given for utilization of sequence reads by the indicated programs. The results obtained during assembly of the genome according to version A (Celera) and version B (Celera and Arachne) in obtaining the first version of the line B10 genome are described in more detail below:

- Version A – assembling the 8× UN and 4× PA reads with sequences of BAC ends using the Celera 5.4 program

The assembled pyrosequenced and BES reads provided 15,667 contigs with a combined length of 197 Mbp, average length of 12,972 bp and N50 of 27,086 bp. The number of supercontigs was 4,173 with a total coverage of 224 Mbp, average length 54,070 bp and N50 2,324,038 bp. Another result of the genome assembly was the identification of contigs of repetitive sequences. These degenerate contigs contain partly assembled repetitive sequences, which could not be uniquely assigned by the Celera program to the remaining sequences. There were 148,607 degenerate contigs with a joint length of over 72 Mbp and average size of 490 bp. In average they were made in 60% of the repetitive sequences.

- Version B stage 1 – Celera – assembling the 8× UN and 4× PA reads after 454 Titanium sequencing using the Celera 5.4 program

The result of the assembly of the reads after pyrosequencing was 15,514 contigs of 195.6 Mbp, with an average length of 12,839 bp, and N50 of 24,714 bp. The number of supercontigs was 4,976 with a total coverage of 197.6 Mbp, average length of 39,799 bp, and N50 of 158,310 bp. The number of degenerate contigs was 160,714 with an average length of 476 bp, jointly encompassing 76.5 Mbp from which, in average, over 61% were recognized as repetitive sequences.

- Version B stage 2 – Arachne – hybrid assembly of results from stage 1 with sequences of BAC ends and 1,781 additional cucumber sequences (see: Material & Methods) using the Arachne program.

As a result of hybrid assembly,16,547 contigs were obtained with a total length of 193.2 Mbp, an average of 12,214 bp and an N50 equal to 23,280 bp. The number of supercontigs was 13,129 with a total length of 323 Mbp, average coverage of 25,865 bp and N50 of 323,092 bp.

**Table 1:** Cucumber genome assembly statistics.

Assembly version	Number of contigs	Average contig length (bp)	Contig N50^a^ (bp)	Total contigs length (Mbp)	Number of scaffolds	Average scaffold length (bp)	Scaffold N50^a^ (bp)	Total scaffold length (Mbp)
454+Sanger – Celera (version A)	15,667	12,972	27,086	197	4,173	54,070	2,324,038	224
454+Sanger – Celera/Arachne (version B step 2)	16,547	12,214	23,280	193	13,129	25,865	323,092	323

a N50 refers to the size above which half of the total length of the sequence set can be found.

The sequence of the genome of the Borszczagowski cultivar (line B10) with the characteristics given in the second row of Table 1 (Celera+Arachne, version B step 2) was deposited in GenBank under No. ACYN01000000 on September 21, 2009. It is also available, together with additional data (i.e. gene predictions, proteins, functional annotation) under the web address http://csgenome.sggw.pl of the Polish Consortium of Cucumber Genome Sequencing.

For genome assembly, a hybrid method was selected which is often used for combining fragments generated by the classical Sanger technique and by next generation sequencing technologies [50], [53]. The detailed characteristics of reads used in genome assembly are provided in, Tables S1 and S2 and Figures S1 A, B. The sequence unique contigs of B10 line genome assembled in each version (A (Celera) 197,5 Mbp and B (Celera/Arachne) 193 Mbp), is similar (Table S3) to both the result of the Chinese 9930 line assembly (185 Mbp) [13] and American Gy14 line (200 Mbp) (Miller et al. Shotgun Assembly of a Repetitive Plant Genome. Sequencing, Finishing & Analysis in the Future. Meeting 27^th^–29th May 2009 Santa Fe, NM, USA 14 (2009)) [54]. In the work of Huang et al., 2009 [13], the assembly was done using the SOAPdenovo software from mostly Illumina reads (68,3× coverage) together with “Sanger” generated genomic library end sequences (3,9× coverage). The American Gy14 line genome was assembled from 454 Titanium pyrosequenced reads: unpaired (24× coverage) and paired (12× coverage) (3 Kbp and 20 Kbp) using the Celera (Miller et al. Shotgun Assembly of a Repetitive Plant Genome. Sequencing, Finishing & Analysis in the Future. Meeting 27th–29th May 2009 Santa Fe, NM, USA 14 (2009); http://sourceforge.net/apps/mediawiki/wgs-assembler/index.php?title=Cucumber_Poster) and the Newbler software [54] (http://www.phytozome.net/cucumber.php). As seen in Table S3, the assembly of the B10 line genome using the Celera Assembler alone with the Titanium reads and BESs (version A), only slightly increased the coverage of supercontigs with respect to that obtained using solely pyrosequenced reads (version B step 1). The coverage was increased by 27 Mbp (224 Mbp obtained for the combination of reads vs. 198 Mbp using second generation reads alone). This may be due to the fact that the Celera assembler mapped only 66.54% of BAC ends on the sequence contigs (Table S4) and assigned the rest to “degenerate” repetitive sequences. The percentage of BESs contained in contigs made by the Arachne program (version B step 2) were 82.04%, from which 81.12% were placed on contigs resulted from next generation reads assembly alone (version B step 1). Comparison of both final assembly versions (A – Celera and B – Celera/Arachne), presented in Table 1, indicated that 190 Mbp of sequence showed a similarity ≥98% between assembled versions of the genome, whereas the share of sequences distinguishing them was 10 Mbp (5.12%). The difference is caused mainly by diverse repetitive sequences recognizing algorithms implemented in both assemblers [52], [55]. Those dissimilar number of used BESs together with the specific algorithms, could also be the reason of: a) differences in unique assembled contigs size (Celera: 197,5 Mbp vs. Celera/Arachne: 193 Mbp), b) the mentioned 10 Mbp of sequences dissimilar between both assemblies versions, c) significant differences of genome size in supercontigs (Celera: 224 Mbp vs. Celera/Arachne: 322 Mbp).

Significantly diverse supercontigs' total length resulted mainly from differences of scafolded and nonscafolded contigs length as well as in length of supercontigs' gaps. Total length of real supercontigs (consisting of at least 2 contigs) in each assembly version is similar (Celera: 218 Mbp vs. Celera/Arachne: 228 Mbp), but Celera scaffolds have about 4.5-times less gaps (12%) and about 2-times more genomic sequences (88%). The Celera real supercontigs contain 97% of total contigs length while supercontigs after Celera/Arachne only 52%. Cross mapping of the chromosome anchored sequences of B (Celera/Arachne) and A (Celera) genomic sequences, demonstrated that about 79% of Celera/Arachne single scaffolds (containing only one contig) (ca. 73 Mbp), were located in gaps of its own real supercontigs (not shown). The above mentioned information suggest that the Celera/Arachne scafolded genome size, should probably sum up to 247 Mbp. It become clear that, in compare to the Arachne, Celera assembler made more compressed assembly by finding more connections between contigs, which resulted in less and longer supercontigs.

In order to assemble the sequences more precisely it appears necessary to first combine the results of the two assembled versions by creating a so-called “reconciliation assembly” [56]. Of the two versions we have chosen the Celera/Arachne assembly (version B step 2) to provide the basic outline of the genome because of the larger (by about 15%) number of BAC ends mapped on sequence contigs (Table S4). Because of containing 2-times longer supercontigs, build (in average) from 2-times more contigs, the genome sequence of version A (Celera) was used to anchor more Arachne contigs (version B) (163 Mbp vs. 80 Mbp) to seven cucumber chromosomes. The proper assembly of the cucumber line B10 genome was confirmed by the assignment of 96.56% of 63035 purified Unigene ESTs (International Cucurbit Genomics Initiative (ICuGI), http://www.icugi.org/cgi-bin/ICuGI/EST/home.cgi?organism=cucumber) and of 6 cucumber genomic sequence clones (BAC/Fosmid) of 372,277 bp with an average similarity of 98%. These values are comparable to those given for the assembly of the genome of line 9930. The genome coverage values do not differ from those reported for other species [57], [58]. Mapping of reads after pyrosequencing on assembled sequence contigs indicated that their average coverage is 14.20×, of which over 98% of the genome sequence length has a coverage of over 3×, and 95% is greater than 5× (Fig. S2). The resulted assembled genome coverage is sufficient to correctly recognize SNP and In/Del polymorphism [50], [57], [59]–[61].

It is worth to note, that despite the use of all ranges of paired end libraries (2 Kbp–10 Kbp, 20 Kbp, 40 Kbp, 140 Kbp) in each of mentioned three cucumber genome projects (B10, 9930 and Gy14), the resulted scaffolds total lengths (Table S3) were only about 200 Mbp, what states for 54% of the flow cytometry estimated cucumber genome size of 367 Mbp [12]. The only aberrance is the Celera/Arachne B10 line assembly supercontigs length (322 Mbp – 88%), though, like it was concluded, it should probably sum up to 247 Mbp (67%). The assembled genome length of the other species' recently published sequencing projects using next generation sequencing (NGS) reads, were close to the flow cytometry's estimated genome sizes i.e. 95% wild strawberry (454, Illumina, Solid) [62], 76% cocoa (454, Illumina) [63], 85% turkey (454, Illumina) [64]. We suppose, that the reasons why the draft cucumber nuclear genome sequences, in all the three mentioned world-wide projects (Polish, Chinese, American), sum up to only ca. 200 Mbp, could be: a) specific distribution of repeated sequences and/or b) underrepresentation of genome in sequencing reads. Though the underrepresentation of genome sequence reads from both capillary and second generation sequencing technologies is well known [65], any confirmation of the proposed specific repeat content distribution would need additional in-deep studies. A this moment, we believe that the use of the long direct 3^rd^ sequencing technologies' reads as well as sequencing of ends coming from very long (about 40 Kbp) paired end libraries (needed to jump through the long rRNA stretches [66]) could facilitate the ongoing work to the finished cucumber genome sequence.

#### Characterization of the genome on the basis of BESs and assembled contigs sequences

The preliminary characterization of the genome performed on BESs and genome contigs gave three values: the percentage content of GC pairs (36.60%), the occurrence of microsatellite repeats and plant repetitive elements.

The participation of the total length of microsatellite sequences (Table S5) was found to be 0.73% in BESs and 0.94% in contigs. Though for the comparison proposes the Table S5 contains different measurements, like microsatellites' length, count, density and mean repeat number, to be the most objective we will report here on % of relative length of SSRs. The most abundant SSRs in BESs were single nucleotide repeats (25.91%), dinucleotides(20.15%), tertanucleotides (19.28%) and trinucleotide repeats (18.62%). The penta- to octa-nucleotide repeats constituted 16.04% of all SSRs. Contigs contained mostly, dinucleotides (24.97%), tetranucleotides (23.36%) and trinucleotides (22.15%). The penta- to octa-nucleotide repeats constituted 19.69% of all SSRs.

When comparing the B10 line genome assembly contigs' microsatellite content and distribution (count, density and mean repeat length (Table S5)) with the previous report by Cavagnaro et al. [54] done on Gy14 cucumber line genome, the results are almost identical. The slight difference in reporting content of di- to tetranucleotides is because of the analysis of mononucleotides in our results. When the mononucleotides were not taken into account the results were practically identical (not shown). The content of plant repetitive elements (Table S6) was evaluated as 48.13% in BESs and 17.82% in contigs. 23.42% of BESs repeats were similar to previously classified plant sequences whereas 23.81% were specific for cucumber (mainly satellite DNA type I, II, III, IV) [67]. Known *versus* specific repeat portions in contigs were 7.73% and 10.09%, respectively. The most abundant group of known repetitive sequences were mobile elements (9.70% in BESs and 6.35% in contigs), ribosomal RNA genes (8.96% and 0.20%), retrotransposons (8.65% in BESs) and small RNA sequences (4.63% and 0.11%).

The comparison of microsatellite content between BESs and contigs' sequences indicates more than 2.5-fold lower participation of mononucleotide repeats in contigs, whereas the share of other multinucleotide repeats is higher in assembled contigs. The content of plant repetitive elements in sequence contigs is on the average 3-fold lower than in BESs. The greatest differentiation of the participation of these elements concerns ribosomal RNA genes (45S and 5S) and small RNA sequences, of which there are respectively 48-, 14- and 42-fold fewer in sequence contigs. Genomic contigs mostly contain unique sequences. This is due to the specifics of automatic assembly algorithms of sequencing reads [68]. Therefore, when the finished genome sequence is not available, the comparisons and characterization of the content of repetitive elements is more appropriate on the basis of long, not assembled reads, like the sequences of genomic libraries e.g. BESs

The share of repetitive plant elements in the assembled line B10 genome is approx. 1.34 lower than their content in line 9930 contigs as described by Huang S. et al. 2009 [13]. This may be due to the additional number of contigs which constitute repetitive sequences in the public genome sequence of line 9930. The number of repetitive plant elements in unique sequences (183 Mbp) of the line 9930 genome determined using the database of repetitive cucumber elements created in this work for B10 line was 16.09% and did not differ from the repetitive element content of assembled B10 line genome.

#### Assignment of contigs to chromosomes

From the set of 2051 markers of the cucumber genetic map (http://cucumber.genomics.org.cn) [69] including 995 SSRs, 1,027 DArTs, and 29 SCARs/RAPDs, the available sequences of 1,883 markers (964 SSR and 919 DArT sequences) enabled the assignment of the assembled genome to specific chromosomes. The unique locations were obtained for 1,665 markers on the genome sequence after version B step 2 (Celera/Arachne) (Table S7), through which 80 Mbp of contigs sequences and 160 Mbp of pseudo-molecule scaffolds were assigned to chromosomes. The genome sequence in version A (Celera) allowed the assignment of 173 correctly placed and oriented pseudo-molecule scaffolds of 207 Mbp length (containing 182 Mbp in sequence contigs), through 1,700 uniquely mapped markers. It is worth to note, that because of the manual anchoring of the Celera scaffolds to the chromosomes using BLAST (instead of Arachne module for Celera/Arachne assembly), and the use of long sequence markers it was possible to choose only the Celera pseudo-molecule scaffolds which were not only placed but also oriented correctly.

The genome sequence obtained in version A, due to the presence of 2-fold longer scaffolds containing 2-fold additional contigs, was used to facilitate the assignment of a larger number of Celera/Arachne sequences (Table S7) to seven cucumber chromosomes. Subsequently as a result of the unique cross-alignment of sequences of both final assembly versions (Table 2), a total length of 163 Mbp of Celera/Arachne contigs as well as 167 Mbp of Celera contigs were finally assigned to chromosomes. That cross alignment with the correctly placed and oriented Celera pseudo-molecule scaffolds enabled also the correct placing and orientation of Arachne/Celera sequences. The above mentioned contigs were assigned through 9358 Celera/Arachne pseudo-molecule scaffolds (300 Mbp) and 169 Celera pseudo-molecule scaffolds (207 Mbp).

**Table 2:** The final result of joint anchoring of genome sequences to chromosomes^a^.

Chromosome number^b^	Number of Arachne contigs	Total Arachne contigs length (Mbp)	Number of Arachne pseudo-molecule scaffolds	Total Arachne scaffolds length (Mbp)	Number of Celera contigs	Total Celera contigs length (Mbp)	Number of Celera pseudo-molecule scaffolds	Total Celera scaffolds length (Mbp)
1 (4)	1519	20.30	1209	34.91	1208	20.87	24	28.60
2 (2)	1480	20.24	1198	34.86	1185	20.81	22	25.16
3 (3)	2411	33.96	1918	60.83	1927	34.89	30	39.09
4 (6)	1974	26.13	1565	47.75	1583	26.81	24	33.16
5 (1)	1633	22.59	1243	45.04	1286	23.18	20	29.98
6 (5)	1736	23.50	1368	45.98	1388	24.05	31	30.31
7 (7)	1125	15.97	857	31.14	879	16.47	18	20.44
Sum	11878	162.73	9358	300.53	9456	167.11	169	206.73
% of assembled sequences	71.78	84.23	71.28	93.04	60.36	84.83	4.05	92.29

a These are the final results of anchoring the genome sequences on chromosomes after cross mapping both of the final versions of the assembly (version A - Celera *versus* version B step 2 – Celera+Arachne) onto themselves and onto the genetic markers.

b Borszczagovski kariotype numbering (brackets contain Chinese Long kariotype numbering).

Genomic contigs of the line B10 were finally assigned to chromosomes on the basis of 1,701 markers, which represents 3.6% fewer markers than used for line 9930 [13]. This difference may be due to the genetic distance between B10 line (European cucumber) and the individuals from the population used for mapping line 9930 (Asian cucumber) [13]. In spite of this difference, 2% and 4.5% more sequences of, respectively, Celera/Arachne and Celera contigs were assigned and the length of pseudo-molecule scaffolds mapped to seven chromosomes was 70% (version B step 2) and 17% (version A) greater than that of line 9930 [13]. The participation of a larger part of the line B10 genome placed on chromosomes, relative to line 9930, both with respect to contigs as well as pseudo-molecule scaffolds, is probably due to B10 having 2-fold longer scaffolds with a greater number of longer contigs and/or the use of other methods of sequencing and other algorithms of sequence assembly.

#### Gene prediction

As a result of *ab initio* gene prediction using GeneMark-ES software [70] on 15,678 genomic contigs with masked repetitive sequences, we obtained 26,587 gene models encoding proteins longer than 50 amino acids. The predicted gene structures (Table S8) contain an average of 5.49 exons each with an average length of 201 bp and introns with an average length of 436 bp. Intergenic sequences have an average length of 3,009 bp. The average length of the coding region is 1103 bp and the average length of the transcribed region is 3,058 bp. The average gene length (including introns) is 4,563 bp.

The sensitivity (Sn) and specificity (Sp) of the gene prediction method was determined on a test set (Table S9).

We also used the GeneMark-ES models to predict genes in the genomic contigs of the Chinese Long cultivar (Table S8). For 12,195 contigs, we obtained 26,442 gene models encoding proteins longer than 50 amino acid residues. The predicted gene structures contain an average of 5.56 exons with an average length of 216 bp and introns with an average length of 434 bp. We observed the following average lengths for intergenic sequences (2,668 bp), coding regions (1,200 bp), transcribed regions (3,178 bp), and the entire genes including introns (4,512 bp).

The average lengths of introns, exons and their numbers are similar for the line B10 and 9930 genomes when comparing gene predictions done in this project and as by Huang et al. 2009 [13] (Table S8), however slight differences can be also observed. Differences are with respect to the number of exons and introns per gene, the length of the intergenic region, and the average length of the translated and transcribed regions. These slight differences between predictions made in this work for genomes of lines 9930 and B10 and the results of Huang S. et al. 2009 [13] could be caused by the different methodologies used for structural annotation and/or genome assembly, as well as the presence of considerable bacterial DNA impurities in line 9930 (Table S8, and Fig. S3 and S4).

In an analysis using the test-set, high values were obtained for sensitivity and specificity of the prediction model (Table S9). These values were similar to those obtained for other eukaryotic organisms, both using demanding programs (for generating a model) and the training set with a large number of cDNA/EST [71], [72] coding sequences as well as using the GeneMark.hmm ES program [70], [73].

#### Functional annotation

Among the 23,190 proposed proteins of line B10 which are longer than 100 amino acid residues, similarities in the GenBank database identified 19,562 of them and 16,944 had informative names (Table S10). A total of 12,463 of the proteins could be assigned to functional groups. In the main Gene Ontology Consortium [74] classification, the following numbers of functional subgroups were identified: 923 BP (Biological Process), 1244 MF (Molecular Function), and 247 CC (Cellular Compoment). In line 9930, out of 23,480 proteins longer than 100 amino acid residues, similarities were identified for 20,361 predictions. Among these there were 17,937 described proteins, and 13,485 of these were assigned to functional groups (Table S10). In the detailed Gene Ontology Consortium [74] classification, the following numbers of functional subgroups were distinguished: 1,023 BP, 1,412 MF, and 266 CC.

The analysis of functional groups indicated that 1,500 proteins from line 9930 (in contrast to only 5 from line B10) had functions specific for bacterial proteins such as: chemotaxis, ciliary or flagellar mobility, enterobacterial common antigen biosynthetic process, flagellum assembly, flagellum organization, gram-negative-bacterium-type cell wall and pilus, spore germination. After careful analysis of the sequences of contigs containing these proteins, 554 contigs (average GC pair content 55.54%) with a high similarity (approx. 85%) to bacterial genome sequences (Fig. S3) were isolated and removed from subsequent analyses. The characteristics of genome sequences before and after removal of sequences representing bacterial contamination are presented in Table S8 and the updated functional statistics of proteins for the purified set of contigs of line 9930 are shown in Table S10. Figure S4 indicates the participation of individual bacterial genera to which the sequences of the assembled contigs of line 9930 (GenBank database under No. ACHR01000000) show similarity.

### Whole genome comparisons between European and Asian cucumber cultivars

#### Analysis of SNPs and INDELs

The alignment of genomic contigs of the cucumber cultivars is presented in Figure S5. Comparative analysis of unique mapped contigs longer than 1000 bp of the European and Chinese lines, 13,428 (187.8 Mbp) and 11,219 (181.8 Mbp), respectively, showed 97.40% similarity of the assembled sequences.

The characteristics of SNP and INDEL polymorphism between compared cucumber genomes are presented in Table S11. SNP polymorphism was generally 2 times higher than INDELs. Uncategorized whole genomic sequences polymorphism frequency was 1/237 bp for SNPs and 1/395 bp for INDELs. It was found that frequency of SNPs and INDELs in exons (1/626 bp (SNP) and 1/1724 bp (INDEL)) is in average 2-times lower than in genes (introns and exons together) (1/403 bp and 1/729 bp) and more than 4-times lower than found in whole genomic contigs sequences. Regions of gene's promoters (−1000 bp upstream translation start), showed polymorphism level (1/260 bp and 1/417 bp) similar to that of uncategorized whole genomic sequences. Beside exons, where the ratio of insertions and deletions is equal, all other parts of the genome sequences have 2-times lower frequency of insertions then deletions in B10 line.

Analysis of INDELs' length distribution (1–2 bp (ultra), 1–10 bp (micro), 11–50 bp (mini), 51–100 bp (midi) – Table S12), showed that in uncategorized whole genome sequences as well as in noncoding promoter regions of B10 line, almost 60% of all insertions and 85% of all deletions are ultrashort (1–2 bp) (INDEL polymorphism will be counted according to the B10 line). Insertions and deletions of micro regions (3–10 bp) taken 25% and 10%, and of mini regions (11–50 bp) taken 15% and 3%, respectively. The longest (midi regions: >51 bp) insertions and deletions, taken 1% and 0.1%, respectively. Unlike above mentioned sequences, exons have almost equal amounts of insertions and deletions (average share of 51% ultra-, 27% micro and 16% mini- INDELs). The slight difference of share were found only for the midi- INDELs. There were 5% of insertions and 8% of deletions. It was also found that exons have 12-times higher frequency of midi-deletions (>51 bp), more than 8-times lower frequency of ultra-deletions and 4-times lower frequency of ultra-insertions than in the rest of the genome sequences.

SNP polymorphism frequency found between two cucumber lines (4.22/1 Kbp), is slightly lower than the one of Arabidopsis thaliana ecotypes (4.68/1 Kbp) [60], [75] and almost 3-times lower than of *Oryza sativa* indica and japonica varieties (31.74/1 Kpz) [76]. The SNP polymorphism frequency in cucumber coding sequences (exons) (1.60/1 Kbp), is almost 2-times lower than in *Arabidopsis* (2,79/1 Kbp) and rice (3/1 Kbp). The cucumber promotor regions are also slightly less polymorphic (3.85/1 Kbp) than in rice (4.61/1 Kbp). The cucumber INDEL polymorphism frequency (<100 bp) found in uncategorized whole genome sequences (2.53/1 Kbp) was almost identical to that of rice (2.66/1 Kbp), but almost 4-times higher than in *Arabidopsis* (0.65/1 Kbp). The differences found in exons are more significant. There were above 2.5-times more insertions and deletions found in cucumber (0.58/1 Kbp) than in rice (0.22/1 Kbp) and 29-times more than in *Arabidopsis*. The INDELs polymorphism frequency found in cucumber promotor regions was almost 2-times higher (2.4/1 Kbp) than in rice (1.28/1 Kbp). The rates of polymorphism found in those three species, seems to mirror the geographical and climate diversity between compared genotypes, their genetic background as well as the share of repeated sequences in the genomes – which are the most variable genome regions.

The most frequent type of sequencing error generated by pyrosequencing introduce artificial insertions or deletions of single nucleotides. This type of error arise when sequencer reads the genome region containing single nucleotides repeats (homopolymer runs) [50], [61], [77]. Those artificial INDELs could cause open reading frame shifts which then affect gene predictions and effect in functional annotation's artifacts [78], [79]. The assembled genome's pyrosequencing reads coverage of 14× is considered to be sufficient for automatic (made during genome assembly) corrections of mentioned above homopolymer errors [50], [61]. The more direct evidence of assembled sequence correctness is that ultra short (1–2 bp) INDELs (those which cause open reading frames shift) frequency was 6-times lower in exons than in other parts of the genome.

#### Analysis of genomic rearrangements

**Figure 1:**
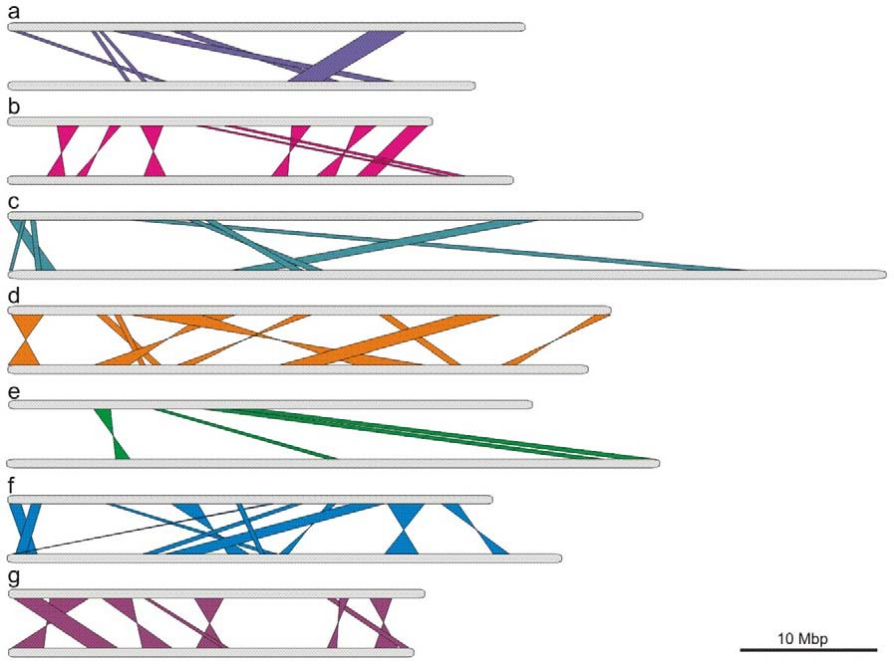
Schematic representation of chromosomal rearrangements between cucumber varieties. Figure shows chromosomal localization of inversions and translocations between genomic sequences anchored on chromosomes I–VII and a comparison between the B10 and 9930 lines (chromosome numbering is according to the Borszczagowski karyotype [80] (brackets contain Chinese Long [67] karyotype numbering)). a - Chromosome 1 (4), b - Chromosome 2, c – Chromosome 3, d – Chromosome 4 (6), e – Chromosome 5 (1), f – Chromosome 6 (5), g – Chromosome 7. The top panel bar represents B10 chromosome with scaffolds, the bottom bar represents 9930 chromosome with. The lines show chromosomal rearrangements between sequences of two genomes.

Besides of the single nucleotide polymorphism, another element of genetic differentiation of lines B10 and 9930 is represented by genomic rearrangements of smaller or larger DNA fragments. Such changes include inversions and translocations, as well as deletions/insertions and duplications, which have accumulated over many generations and enabled the plants for better adaptation to new growth conditions. This has been demonstrated for primitive organisms as well as higher plants [16]–[19]. Most rearrangements observed between genotypes of two cucumber cultivars are of the inversion/translocation type (Fig. 1 (schema) and Fig. S6 (detailed comparison)). The translocations and inversions affect all chromosome and involve segments even as large as 5 Mbp. Translocations are mostly between the inner chromosome regions and the ends of the chromosome arms, so it was probably the way to make some “desired” genes more transcriptionaly active than other “not needed” genes. These rearrangements may contribute to the differentiation of the two cucumber lines and thus could be a novel aspect of the evolutionary adaptation mechanism.

#### Analysis of functional groups differences

The above structural differences can lead to changes in gene quantity between analyzed cucumber cultivars. To check if it is true, the functional annotation results were compared between cultivars. As shown in Figure S7A–C and in Dataset S3, the intra-species differences between both lines as well as between species are minimal. In deep comparative functional analysis were done for 38 functional groups (16 BP, 18 MF, 4 CC), which show at least 2-fold higher number of genes in one of the two genotypes. Those functional groups in B10 and 9930 lines, contained 126 (ca. 1% of GO genes) and 96 genes (0.78% GO genes), respectively. The analysis indicates (Table 3 and Table S13) that in line B10, there is a larger number of genes involved in photosynthesis, sugar metabolism, respiration, regulation of gene expression and chlorophyll degradation, as well as more genes involved in assimilation of nitrogen in the form of ammonium ions. The Asian cucumber variety, on the other hand, is characterized by having more genes involved in regulation of tolerance to photo-oxidative stress and high temperatures.

**Table 3:** Functional differences between cucumber lines B10 (European) and 9930 (Chinese).

Process	B10 line^a^	9930 line^a^	Environmental conditions
Photosynthesis	+	−	Temperate climate zone of northeast Europe: chilling temperatures, lower sunlight intensity
Sugar metabolism	+	−	
Respiratory complex	+	−	
Regulation of gene expression	+	−	
Chlorophyll degradation	+	−	
Nitrogen fixation in the form of ammonium ions	+	−	Continuous higher CO_2_ emission in Europe from the beginning of the industrial era and the 90's of 20^th^ century – reduced fixation ability of nitrate ions
Response to oxidative stress	−	+	Subtropical climate zone of southeast China: higher seasonal average sunlight intensity, UV-B irradiation and temperature
Response to high temperature	−	+	

a + or − indicate which line has respectively significantly higher or lower number of genes involved in each process.

### Comparative analysis of gene promoters between species

#### Distribution of ABREs, DREs and EREs in promoters of genes of *C. sativus* line B10

A total of 20,745 promoter sequences (−1,000 bp from the ATG start codon) of genes of *C. sativus* line B10 were analyzed with respect to the occurrence and distribution of ABREs, DREs and EREs. It was found that 1,294 ABREs, 4,118 DREs and 449 EREs occur in 1158, 3500 and 438 gene promoters, respectively. Statistical analysis indicated an enriched content of the analyzed CREs in gene promoters of *C. sativus* line B10 (Table S14). The enrichment factors for ABRE, DRE and ERE are 1.27, 0.95 and 2.09, respectively. Promoter sequences of the analyzed genes were divided into 50 nucleotide fragments. In each fragment, the content of the analyzed CREs was calculated. An increased density of the analyzed fragments was identified at a distance of about 300 bp from the start codon ATG (Fig. S8).

#### Relative content of ABREs, DREs and EREs in promoters of genes of *A. thaliana*, *P. trichocarpa*, *O. sativa* and two *C. sativus* lines: B10, 9930

The occurrence of specific CREs and combinations thereof in gene promoters may explain the putative correlation between these elements and the specific plant responses to biotic and abiotic stress and as such, will determine the Darwinian fitness of a plant under chilling or freezing or drought conditions. For this reason we identified all genes in *A. thaliana*, *P. trichocarpa*, *O. sativa* and in two *C. sativus* lines: B10 and 9930, whose promoter sequences contain specific CREs (for detailed analysis of CREs in *C. sativus* line B10 promoters see Table S14 and Fig. S8). We calculated the relative numbers of ABREs, DREs and EREs *per* genome (Table 4). The relative percent content of each of the analyzed CREs was calculated according to the formula = ((ABRE+DRE+ERE)/CRE)×100. In the promoters of all identified genes of the analyzed plant species, the occurrence of the DRE element is approximately 70%. The content of the ABRE element in the promoters of genes from *A. thaliana*, *C. sativus* and *P. trichocarpa* is similar and is approximately 22%, whereas in *O. sativa* it is only 8%. The number of ERE elements in the promoters of *A. thaliana*, *C. sativus* and *P. trichocarpa* genes is approx. 8%, whereas in *O. sativa* the content of this CRE is 18% (Table 4).

**Table 4:** Relative content of ABREs, DREs and EREs in promoters with CREs in *A. thaliana*, *O. sativa*, *P. trichocarpa*, and *C. sativus* lines B10 and 9930.

	ABRE			DRE			ERE		
	%	P-value	Average	%	P-value	Average	%	P-value	Average
*A. thaliana*	24.8%	2.86E-05	1.1763	67.7%	1.33E-04	1.2098	7.4%	7.42E-06	1.037
*C. sativus* line B10	22.1%	2.47E-05	1.1174	70.3%	1.08E-04	1.1766	7.7%	5.42E-06	1.0251
*C. sativus* line 9930	22.4%	2.47E-05	1.1385	69.4%	1.09E-04	1.1885	8.2%	5.46E-06	1.0509
*O. sativa*	8.0%	5.30E-05	1.1121	73.7%	3.45E-04	1.6561	18.3%	3.48E-05	1.2503
*P. trichocarpa*	19.1%	2.80E-05	1.1234	70.8%	1.27E-04	1.3172	10.1%	7.01E-06	1.1154

The relative percent content of each element is calculated from the formula ((ABRE+DRE+ERE)/CRE)×100%.

The above results indicate that *A. thaliana* plants which are capable of overwintering in the vegetative form (as a green rosette), have the highest relative number of ABREs in their promoters and the lowest numbers of DREs and EREs relative to the remaining analyzed species and lines. The situation is different in *C. sativus* and *P. trichocarpa*. These plants do not overwinter like cucumbers, nor do they overwinter without leaves, like poplar trees. In the genome of *O. sativa*, a subtropical plant, which is not adapted for growth in ecosystem with chilling or freezing temperatures relative ABRE elements content is the lowest among all analyzed species. *O. sativa* is however adapted to others stresses such as root hypoxia and associated with it water uptake problems. Ethylene and ROS homeostasis in plant cells is controlled by a number of genes that regulate formation of lysigenous aerenchyma, light acclimatization and defense responses [21], probably therefore has the highest number of ERE and DRE and the lowest ABRE elements in its genome. However orthologous groups of genes encoding *trans*-elements that interact with these CRE are highly conserved [81] (Fig. S11, S12, S13, S14). The comparison of the occurrences of the three analyzed CREs in the promoters of two cucumber lines also showed significant differences. Promoters of genes in line 9930 are characterized by increased ABRE and ERE content and decreased DRE content, relative to the B10 cucumber line.

#### Functional classification of *A. thaliana*, *P. trichocarpa*, *O. sativa*, and *C. sativus* line B10 genes containing ABREs, DREs and EREs in their promoters

Genes of the analyzed species were classified according to function and cellular localization, into appropriate gene ontology (GO) groups: GO Cellular Component, GO Molecular Function and GO Biological Process. In the promoter sequences of the genes of each group, the occurrences of ABREs, DREs, EREs and the combinations ABRE+DRE, ABRE+ERE, DRE+ERE, and ABRE+DRE+ERE were determined for *A. thaliana*, *O. sativa*, *P. trichocarpa*, and *C. sativus* line B10. The analysis indicated differences in the number of chloroplast genes among the analyzed species (Fig. S9C). Genes belonging to this group are the most numerous in *A. thaliana* and *P. trichocarpa* and constitute approximately 10% of the pool of all analyzed genes for these species. Among the *A. thaliana* chloroplast genes, a significant increase was observed for the combinations ABRE+ERE (14%) and ABRE+DRE+ERE (16%) in their promoter sequences. The number of genes encoding proteins linked to the cell wall is decreased in *C. sativus* (0.5%), relative to genes of this class in the other species investigated (2–2.8%). In all analyzed species a preponderance of genes encoding proteins with hydrolase activity, which contain the combinations ABRE+ERE+DRE, DRE+ERE and ABRE+ERE in their promoters was observed (Fig. S9B). In *C. sativus*, on the other hand, we identified an increase in the number of genes encoding proteins with kinase activity, which contain the combinations ABRE+DRE+ERE and ABRE+DRE in their promoters.

The analysis of *C. sativus* line B10 genes linked to signal transduction indicated a distinct preponderance of genes containing the combination ABRE+DRE+ERE (7.6%) in their promoter sequences with respect to genes represented in this category in the remaining species (Fig. S9A). On the other hand, considerably fewer genes (approximately 2%) were identified as being linked to stress responses in line B10. Genes belonging to this class are the most highly represented in the three remaining species. *P. trichocarpa* contains EREs in 11.8% of its promoters, and the combination ABRE+ERE in 11.7% of its promoters. *O. sativa* contains ABREs in 8.7% of its promoters, the combination ABRE+DRE in 7.9% of its promoters, and the combination ABRE+ERE in 8.6% of its promoters. *A. thaliana* contains the combination ABRE+ERE in 8.3% of its promoters, and the combination ABRE+DRE+ERE in 8.5% of its promoters.

Among the genes involved in plant responses to biotic and abiotic factors, the most common combinations of elements in the promoters were ABRE+ERE in *A. thaliana* (8.6%) and in *O. sativa* (6.9%), ABRE+DRE+ERE (7.6%) in *C. sativus*, and ABRE+DRE (5,1%) in *P. trichocarpa*.

#### Functional analysis of genes containing ABREs, DREs and EREs in their promoters

**Figure 2:**
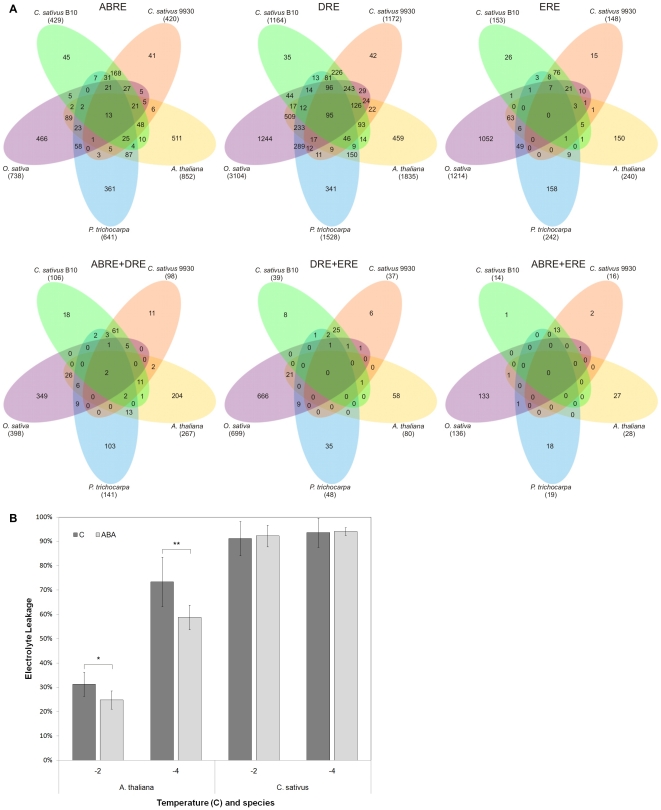
Comparison of CRE content in orthologous genes and freezing tolerance tests of non-acclimated *A. thaliana* and *C. sativus* seedlings after abscisic acid (ABA) treatment. (*A*) Venn diagrams presenting the number of common and different orthologous genes' groups (5,971) with respect to the occurrences of ABREs, DREs, EREs and their combinations in promoters in *A. thaliana*, *P. trichocarpa*, *O. sativa* and lines B10 and 9930 of northern European and Chinese *C. sativus* varieties. Total number of orthologous groups with specific CRE or combination of CREs is given in brackets. (*B*) Freezing tolerance tests of non-acclimated *A. thaliana* and *C. sativus* plants after ABA treatment. Leaves were frozen in different temperatures and cellular damage was assessed by measuring electrolyte leakage. Statistically significant difference was assessed by Student's t-test – n = 5, * p-Value<0.05; ** p-Value<0.005. Error bars indicate standard deviations. C – control non-treated plants; ABA – abscisic acid treated plants.

In order to determine which groups of genes in different species and lineages are regulated by the same CREs, and the same combinations of CREs and to analyze evolutionary changes in promoter sequences, we identified 5971 groups of orthologous genes from *A. thaliana*, *P. trichocarpa*, *O. sativa* and *C. sativus* line B10 and 9930 (Dataset S1) and we compared the genes which contain ABRE, DRE, ERE elements, the following combinations of two elements: ABRE+DRE; ABRE+ERE; and DRE+ERE in above mentioned species and lineages (Fig. 2A and Dataset S1). The functional classification of genes with above mentioned combinations of CREs is provided in Fig. S9.

We identified 429, 420, 511, 361 and 738 groups of orthologous genes with promoters containing ABREs in *C. sativus* line B10, *C. sativus* line 9930, *A. thaliana*, *P. trichocarpa* and *O. sativa*, respectively. For DREs, we identified 1,164; 1,172; 1,835; 1,528 and 3,104 groups of orthologous genes with promoters containing these CREs in *C. sativus* line B10, *C. sativus* line 9930, *A. thaliana*, *P. trichocarpa* and *O. sativa*, respectively. For EREs, we identified 153, 148, 240, 242 and 1,214 groups of orthologous genes with promoters containing these CREs in *C. sativus* line B10, *C. sativus* line 9930, *A. thaliana*, *P. trichocarpa* and *O. sativa*, respectively. For the combination of elements ABRE+DRE, we identified 106, 98, 267, 141 and 398 groups of orthologous genes with promoters containing these CREs in *C. sativus* line B10, *C. sativus* line 9930, *A. thaliana*, *P. trichocarpa* and *O. sativa*, respectively. For the DRE+ERE combination, we identified 39, 37, 80, 48 and 699 groups of orthologous genes with promoters containing these CREs in *C. sativus* line B10, *C. sativus* line 9930, *A. thaliana*, *P. trichocarpa* and *O. sativa*, respectively. For the combination of elements ABRE+ERE, we identified 14, 16, 28, 19 and 136 groups of orthologous genes with promoters containing these CREs in *C. sativus* line B10, *C. sativus* line 9930, *A. thaliana*, *P. trichocarpa* and *O. sativa*, respectively.

Among the analyzed promoters of groups of orthologous genes containing ABREs in *A. thaliana*, *P. trichocarpa O. sativa* and two lines of *C. sativus*: B10 and 9930, only 13 were found to be common in these plant species (Fig. 2A). 95 groups of orthologous genes with promoters containing DREs were conserved in all analyzed species and lineages. In *A. thaliana*, *P. trichocarpa O. sativa* and two lines of *C. sativus*: B10 and 9930, among groups of orthologous genes containing ABRE+DRE combination in their promoters, 2 were common for the analyzed species (Fig. 2A, Dataset S1). These genes encode the following proteins: putative late embryogenesis abundant (LEA) and putative protein phosphatase 2C We have not identified any conserved groups of orthologous genes with promoters containing EREs and the following combinations of two elements: ABRE+ERE; and DRE+ERE in above mentioned species and lineages (Fig. 2A, Dataset S1).

The analysis of the frequency of occurrence of mutations between genes (introns and exons) and their promoters within the two analyzed cucumber lines has indicated that both SNPs, as well as INDELs occur approximately 3 times more frequently in promoter regions than in gene sequences (Table 5). Promoters of selected orthologous genes of two *C. sativus* lines were compared using PlantCARE program [82] and significant changes were observed in promoter sequences, which changed CRE combinations (Fig. S10).

**Table 5:** Analysis of the frequency of occurrence of mutations in exons and introns and their promoters with different combinations of CRE in *C. sativus* lines B10 and 9930.

	SNPs		INDELs	
	Promoter	Exons+Introns	Promoter	Exons+Introns
No. of Mutations^a^	14.89^***^	4.60	3.87^***^	1.34

a Values indicate number of mutations per 1000 bp only in genes containing ABRE, DRE and ERE. Statistically significant difference was assessed by Student's t-test - *** p-Value<0.001.

In order to confirm *in silico* prediction of different regulatory responses in different plant species to the same hormone we perform experiments with abcisic acid (Fig. 2B). Non-acclimated *Arabidopsis* and cucumber seedlings growing in ambient laboratory conditions were treated with 200 µM abscisic acid solution for 3 days and tested for freezing tolerance. Ion leakage after freezing to −2 or −4°C was significantly lower in *Arabidopsis* than in cucumber seedlings (Fig. 2B).

#### Characterization of *C. sativus* proteins linked to abiotic stress response

We analyzed genes encoding proteins from the LEA group (***L*** *ate* ***E*** *mbryogenesis* ***A*** *bundant*). In *C. sativus* line B10, only 26 genes of this type are present. For comparison there are 51 genes of this class in *A. thaliana* [83] and 28 in *P. trichocarpa*. In *C. sativus* line B10, 9 of the LEA genes (34.6%) had ABREs and 8 had DREs (30.8%) in their promoter sequences. In *A. thaliana*, 32 genes of this class had ABREs (56.1%) and 34 had DREs (59.6%) in their promoter sequences. In *P. trichocarpa* 12 genes of this class had ABREs (42.9%) and 10 had DREs (35.7%) in their promoter sequences. These results may suggest that the decreased tolerance of cucumber to unfavorable environmental conditions (e.g. cold temperatures and water deficiency) is linked not only to the low number of LEA genes but also has a statistical effect on the relatively reduced presence of ABREs and DREs in their promoters. Cucumber sensitivity to stress probably also causes a reduction in the number of genes encoding scavengers or reactive oxygen species. In line B10 there are 116 such genes, whereas *A. thaliana* has 148. Only 3 groups of genes were present in similar numbers in *C. sativus* line B10 and *A. thaliana*. Among these genes are the monodehydroascorbate reductase genes, glutathione reductases, and catalases. The remaining groups (superoxide dismutases, ascorbate peroxidases, dehydroascorbate reductases, glutathione peroxidases, NADPH oxidases, peroxiredoxins, thioredoxins and glutaredoxins, were represented by a smaller number of genes in cucumber than in *A. thaliana*. We also described putative transcription factors representing AP2_EREBP and bZIP families from *C. sativus* line B10 and *C. sativus* line 9930, respectively (Dataset S2). We identified putative representatives of AREBs (Abscisic Acid–Responsive Element Binding Protein), ERFs (Ethylene-responsive Element-binding Factor) and DREBs/CBFs (Dehydration-responsive Element-binding proteins) (Fig. S11, S12, S13, S14).

## Discussion

The draft genomic sequence of the North-European Borszczagowski cultivar of the cucumber *C. sativus* (line B10), provided herein, and the comparative genomics studies presented, has brought not only applied genomic research of cucumber but mainly basic research on plants to a new level. A quality draft of a highly repetitive plant eukaryotic genome was obtained by using a hybrid method of assembly utilizing the ends of clones of a BAC library combined with paired and unpaired 454 Titanium pyrosequencing fragments summing up to 12× coverage.

The assembled portion (53%) of the cucumber nuclear genome sequence (without gaps), has more than 14× coverage with 454 Titanium reads. As it became clear from the ultralength (1–2 bp) INDELs polymorphism analysis made in this work, and according to the other researchers [50], [61], such a coverage rate, is sufficient to eliminate pyrosequencing specific errors. Because the presented result of cucumber B10 line genome assembly is practically identical to those of American Gy14 line (36× coverage) [54] http://www.phytozome.net/cucumber.php) and Chinese 9930 line (70× coverage) [13], it seems that chosen whole genome sequencing method was the most effective one to get the first quality draft sequence of cucumber genome. By cross mapping each other assembly versions (Celera vs. Celera/Arachne), it was possible to anchor more Celera/Arachne contigs (the most genome representative) to the chromosomes. The assembled genome served also for de-novo gene prediction, functional annotations of genes and comparative genomics studies. The average contig size of 12 Kbp and especially almost 95% of genome contained in contigs longer than average gene length of 4 Kbp, gives the possibility to discover almost all protein coding genes.

The Darwinian fitness of individual species and/or lines could be determined by a species-specific set of genes and also by the whole regulatory network formed by the specific activation/deactivation of TRFs, which in turn interact with specific CREs residing within gene promoters.

We observed that the European cultivar in comparison to Chinese Long cultivar (Table 3 and Table S13a) has increased number of genes involved in photosynthesis, respiration, sugar metabolism, chlorophyll degradation and regulation of gene expression. This might indicate for adaptation to the temperate European climate conditions (lower average light intensity and grow temperatures). On the other hand Chinese Long cultivar has more genes related to photooxidative stress tolerance (UV and high light irradiation), higher non-optimal temperatures tolerance (e.g. heat shock proteins) and pathogen attack resistance (e.g. peroxidases and glycoproteins), therefore is better adapted to the subtropical climate conditions. We have also found that in line B10 of the European cucumber cultivar, a much larger number of genes take part in ammonium ion assimilation. This is expected to compensate for the limited ability to assimilate nitrate ions [84], [85], probably as a result of higher levels of continuous CO_2_ emission in Europe till the 90's of the 20^th^ century. Industrial era in Europe started at the end of 18th century, but taking into consideration that significantly higher carbon dioxide atmospheric emission started together with wide availability of internal combustion engine at the end of 19th, it could be concluded that mentioned adaptive genomic changes arose in about 100 generations. Unicellular yeast, needs only about 2–3-times less generations to acquire new genomic adaptations [19]. It seems like the time needed by multicellular cucumber plants to adapt to new environmental conditions is extremely short and constitute the new evidence of “evolution in action”. Recently the similarly fast environmental adaptation was reported for Atlantic tomcod (*Microgadus tomcod*) living in Hudson River, NY, USA [86].

Rearrangements of inversions and translocations type which differ two cucumber genotypes, growing in extremely diverse environmental conditions, could have arose as a result of evolutionary adaptation to specific conditions. Functional comparative analysis between cucumber genotypes, showed that 9930 line genome contains 4-times more copies of peroxidase genes and 3-times more copies of two dehydrogenase genes, which are crucial for oxidative stress resistance in plants [17]. Like it was reported in pathogen *Cryptococcus neoformansand* and in yeast, oxidative abiotic stress caused by high temperature, UV light or hydrogen peroxide result in wide range of chromosomal rearrangements (translocations, deletions, inversions, amplifications, chromosome fusions or aneuploidy), which are stabilized over generations and lead to resistance's acquisition [18], [87]. It was shown in cucumber, that UV-B radiation leads to higher ploidy and bigger size of cells surrounding leaves' trichomes. Additionally the higher expression of peroxidase and dehydrogenase genes required for UV radiation's protection, was noted [17]. In turn, the B10 line increased number of genes responsible for effective energy management, under relatively low temperature and low sunlight intensity conditions, may be the result of chromosomal rearrangements and amplifications of specific genome regions, caused by the necessity to adapt to a given environment. This has been demonstrated in yeast maintained for many generations under conditions of lower availability of nutrients, in particular glucose [14], [19], [88]), and in flax in response to low temperatures [20].

The structural differences leading to changes in gene copy numbers were showed recently in corn between 33 genotypes [89]. The rearrangements (CNVs – copy number variations and PAVs - presence absence variations) were related to the acclimations of the genotypes to different environmental conditions. Recently, large (1 Mbp) chromosomal rearrangements (mostly inversions and translocations) between 17 human genomes sequences were reported. Detection of those extensive rearrangements was enabled by sequencing of 14 million fosmid (40 Kbp insert) library ends [90]. The other work, reported occurrence of large chromosomal duplications in genomes of breast cancer cells. That finding was achievable because of the use of new methodology named array painting [91]. Like it was noted by Swanson-Wagner et al., 2010 [89], till now it was believed, that the same species' organisms should have similar genomes. On the other hand, authors of the recent findings of extensive structural genome differences between whole human genomes or in breast cancer cells, remark that discovery of such genomic changes was not earlier possible, because the broad used methods like FISH (fluorescent in-situ hybridization), CGH (comparative genomic hybridization), SNP microarrays or genome resequencing, do not give such a possibility [90], [91]. It is important to note that in the other cucumber genome project (Gy14 line) the rearrangements were not reported till now. As it is written at the web address http://www.phytozome.net/cucumber.php, the “comparison with the genetic map of cucumber supports the long-range accuracy of the assembly”. We believe that the main reason why they had not found any rearrangements was the use of genetic map containing the markers and their locations common for both Chinese and American background as it cames out from paper of Ren et al. [69]. Such a methodology assumed that the rearrangements are not possible between different genotypes of the same species. This assumption is not only conflicting with our results but also with the very recent paper by Zhao et al. [66] where a large rearrangements of satellite and rRNA genes were reported between *Cucumis sativus* var. sativus and *Cucumis sativus* var. hardwickii.

Regarding to the above comment, it seems that the discovery of global genomic changes (rearrangements) between two cucumber genomes, were possible only because of availability of two genomes sequences generated de-novo together with the use of diverse genome background genetic map.

The differences, we identified by comparing the genomes of two cucumber cultivars at the levels of chromosomes and genes, suggest that specific environmental and evolutionary adaptations of line B10 and line 9930 have occurred in response to the temperate Northern Europe and subtropical southeast Asia climate conditions, respectively, which are different then the Himalayan climate of its origin [92].

Reported in this work, extensive and numerous interchromosomal rearrangements between two cultivars of the same crop species is the novel evidence. It should also be noted, that beside such rearrangements, all of the genotypes are fertile. Though the observed genome rearrangements need further and deeper analysis, it could be concluded that those whole chromosomes shifts could represent adaptive changes, because are accepted and heritable. It could be also supposed that the view of evolutionary genomic adaptations emerging from the previous comparative genomics studies based on i.e. resequencing or hybridization data, is at least only partial and could lead to erroneous conclusions about the real mechanisms of organisms adaptive changes.

Differences in expression of orthologous genes in the two closely related species *D. melanogaster* and *D. simulans* can be explained in most cases by the loss or gain of CREs in promoter sequences [93]. Furthermore, Kasowski with colleagues (2010) [94] showed that binding sides of RNA polymerase II and nuclear factor κB differed significantly between human individuals. Binding differences were linked to SNPs and were often correlated with altered gene expression in humans [94]. Zhang with colleagues (2004) [95] reported that freezing-sensitive tomato plants posses a functional CBF/DREB response pathway, but CBF regulon differs considerably from that of freezing-tolerant *A. thaliana*. Observed differences can be explained by species-specific distribution of functional DRE/CRT elements within promoters of orthologous genes, or by mutations in proteins required for CBF/DREB activity. Moreover, transcriptional variation of auxin responses in seven natural accessions of *A. thaliana* was observed recently by Delkler with colleagues (2010) [96].

It was shown that CRE turnover and shuffling [97], and transcriptional rewiring [98] often occurs in genomes of animals and yeasts over evolutionary time. However, in some cases these alterations do not affect gene expression [97]. In our work we also observed substantial differences in CRE content between all analyzed species and varieties. For example we found significant change in ABRE and ERE content between the genes of *O. sativa* and the other species. This may be partly explained by the higher GC content in *O.* sativa [99] and/or the role of ethylene in adaptation to root hypoxia conditions [21]. We also identified significant differences in CRE distributions (Fig. 2A, Fig. S10 and Dataset S1) between the two lines of *C. sativus* (B10 and 9930). Moreover, we also examined the genes with ABRE, DRE and ERE in their promoters to determine if they belong to the same functional groupings in the analyzed species, we found that only a small fraction of the groups of orthologous genes with the highest sequence similarity in *A. thaliana*, *O. sativa*, *C. sativus* (B10 and 9930) and *P. trichocarpa* have the same CRE profiles in their promoters (Fig. 2A, Dataset S1). These results suggest that, each species and/or varieties has a specific profile of CRE content in gene promoters which actually determines specific changes in gene expression profiles (regulons) selected in response to the similar changes in environmental conditions (e.g. not optimal temperatures or water uptake limitations). We showed that non-acclimated *Arabidopsis* seedlings displayed better freezing tolerance after ABA treatment, while cucumber did not. This could be due to species-specific set of genes controlled by both ABRE and DRE and ABA specific response in the above species (Fig. 2A). Our ABA-treatment experiments (Fig. 2B) together with *in silico* analysis of CRE shuffling (Fig. 2A) explains why *C. sativus* is much more susceptible for cold and chilling stresses than *A. thaliana*. The large changes in sequences of promoters of orthologous genes in *C. sativus* are due to approximately a 3-fold greater number of SNPs and INDELs in promoters relative to exons and introns of the same gene and genotype (Table 5). In *P. trichocarpa* the number of polymorphisms was also found to be about 2-fold higher in non-coding sequences than in exons [100]. This indicate flexibility of gene' promoter sequences [101], [102]. Though our results still need to be confirmed in future experiments, these could indicate that rapid adaptation of a line or variety to new environmental conditions may take place at the level of the regulation of gene expression by changing interactions in a transcriptional network without loss of functionality of particular genes. It is possible that due to the large variability in promoter sequences (CRE shuffling) new lines/varieties may be formed to adapt to new ecological niches.

In conclusion, adaptation of plants to new environmental conditions may occur on the level of changes in gene dosage and gene quality, but the rapid adjustment probably occurs by rewiring of gene regulatory networks driven by CRE shuffling. We suggest that through evolution, eukaryotic organisms have been equipped with a high degree of freedom with respect to the variability of promoters, in terms of regulatory elements, and chromosomal rearrangements that allow for formation of new lines/varieties and species adapted to new ecological niches.

## Materials and Methods

### Plant material

The plant material was the highly inbred (20 generations) monoecious B10 line of the Borszczagowski cucumber, an old field cultivar. The plants were cultivated in a greenhouse during the summer. Material for analysis was obtained from plants cultivated in a greenhouse under controlled photoperiod conditions 16 h/8 h day/night, at a temperature of 25°C–27°C during the day and 18°C–20°C at night. The intensity of the light was 1500 µmol (quantum) m^−2^·s^−1^. Young leaves with a surface area of 2–3 cm^2^ were collected early in the morning directly after 24 hours of darkness, frozen in liquid nitrogen and stored at −74°C. DNA for genomic sequencing was isolated using the GenElute Plant Genomic DNA Purification Kit as recommended by the producer (Sigma Aldrich, St. Louis, MO, USA).

### BAC library

The clones of long cucumber genome fragments used in this work were derived from two BAC libraries, both prepared in the pCC1BAC vector. Among 33,792 available clones 3,072 were amplified in *E. coli* EPI300 cells [103] and the remaining clones were amplified in *E. coli* DH10B cells (Amplicon Express, Pullman, WA, USA). Genomic DNA of clones in EPI300 was previously treated with the *Hind*III restriction endonuclease. The remaining inserts were derived from cleavage with *Mbo*I and were ligated into the vector cleaved with *BamH*I.

### BES sequences

A total of 33,792 BAC clones were used for analysis giving in effect 67,584 BES sequences which were sequenced by the Sanger method using an ABI 3730xl capillary sequencer (Beckman Coulter Genomics, formerly Agencourt Bioscience Corporation, Danvers, MA, USA) and standard M13 Forward and M13 Reverse primers.

Purification of 67,584 BESs to filter out the low quality sequences and the vector and bacterial sequences was performed using Lucy v1.20 [104] and BLAST [105] software. To remove non-nuclear sequences from further analysis, the BES sequences were aligned (using BLAST) to the cucumber chloroplast genome sequences (AJ970307 [106]) and to the set of plant mitochondrial sequences available in GenBank.

### Genome sequencing

Sequencing of BAC library end sequences (BES) were done by the Sanger method using an ABI 3730xl capillary sequencer (Beckman Coulter Genomics, Danvers, MA, USA) and standard M13 Forward and M13 Reverse primers. The whole genome shotgun sequencing was performed by the pyrosequencing method using XLR Titanium reagents (454 Life Sciences, a Roche company, Branford, CT, USA). Sequencing of random fragments described as UNpaired (UN) given 8× genome coverage and of fragments described as PAired ends (PA) produced 4× genome coverage with an average distance of a pair of genomic sequences of 3 Kbp. Paired fragments were connected to each other by a so-called FLX type linker (44 bases) with the sequence 5′-GTTGGAACCGAAAGGGTTTGAATTCAAACCCTTTCGGTTCCAAC-3′. Preparation of the fragment libraries was performed according to the procedures of 454 Life Sciences. Titanium read lengths were characterized after processing by the sffinfo script from the Newbler ver. 2.0.00.20 package (454 Life Sciences) and, for paired libraries, rejecting the linker sequences using the script sff_extract 0.2.3 (http://bioinf.comav.upv.es/sff_extract/index.html).

### Additional cucumber sequences

In addition to all of the above mentioned sequences, we used 1,781 paired and unpaired Sanger generated (Beckman Coulter Genomics, Danvers, MA, USA) (accession numbers: ET203886–ET203889, FH937590–FH937636, FI136294–FI136299, FI160638–FI160866, ES882265–ES883262) sequences which found to be polymorphic among near isogenic lines of cucumber differing in sex phenotype [107]–[109].

### Genome assembly

The genome sequence was obtained after hybrid assembly by a combination of the Celera Assembler ver. 5.4 [52] and Arachne ver. 3.0 [110] programs. The version A assembly was done with Titanium and Sanger reads using the Celera program. The version B hybrid assembly was done by Arachne program using the sequence contigs obtained from assembly of solely pyrosequenced reads by the Celera program (version B step 1) together with BES sequences. Prior to the Arachne assembly (version B step 2), the version B step 1 sequence contigs were cut into fragments of an average length of 800 bp overlapping by 100 bp.. Moreover, information about the Celera scaffolds was maintained in the form of “artificial” paired ends (forward and reverse) with an average length of 800 bp.

### Characterization of the genome on the basis of BESs and genomic sequences

To identify and classify microsatellite sequences, MISA software (http:// http://pgrc.ipk-gatersleben.de/misa/misa.html) was used taking into consideration sequences of mono- to octanucleotides with a minimal length of 12 nt and the minimal number of 3 repeats units. Repetitive plant elements were identified and classified on the basis of aligning BES sequences to the Michigan State University database of Repetitive Plant Sequences [111]. Similarities were analyzed by the tBlastx algorithm with the threshold value 1e-05. The database of repetitive sequences in the cucumber genome was created *de novo* using the RepeatModeler program (http://www.repeatmasker.org/RepeatModeler.html) on the basis of nuclear BES sequences and the repeat database Repbase Update [112]. Masking of repetitive sequences was performed using the RepeatMasker program (http://repeatmasker.org). The above-mentioned method of analyzing microsatellites and repetitive plant elements was also used for genomic contigs.

### Mapping genome on chromosomes

In order to map the line B10 genomic sequences on seven cucumber chromosomes 1,883 available sequences of markers together with the information about their positions on the genetic map (http://cucumber.genomics.org.cn), [69] were used. The mapping was performed using the BLAST (blastn, 1×10^−20^, >30% marker coverage, >90% similarity) and specific Arachne modules on sequences obtained after assembly versions A and B. The cross-aligment of sequences from version A and B, done with the MUMmer 3.22 [113] program, facilitated proper assigment (correct position and orientation) of both assembly version contigs to chromosomes.

### Gene prediction

The small number of available full transcripts (mRNA) which, as of February 2010, was only about 450 or even ESTs (approximately 6500), does not enable us to perform a supervised training for conventional *ab initio* gene finders [71]. Therefore, in this work, we used a novel unsupervised training approach implemented in the GeneMark-ES software. In this method, the un-annotated assembled genome is used as the program input to generate in iterations the algorithm final parameters that are used to obtain gene predictions [70], [73]. The cucumber coding sequences available in databases were used only to check the correctness of the proposed gene structures.

Parameters of the *ab inito* gene finder were determined by unsupervised training on the assembled *C. sativus* contigs by the GeneMark-ES [70], [73] program. We used 3,008 contig sequences longer than 20,000 bp with a joint length of 109 Mbp; repetitive and TE sequences in these contigs were masked. *Ab initio* predictions made by GeneMark-ES were verified using 63,035 cucumber Unigene EST sequences (http://www.icugi.org/) and 422 cDNAs (http://www.ncbi.nlm.nih.gov), which were purified from vector contaminations by the SeqClean program (http://compbio.dfci.harvard.edu/tgi/software/). To create a test set we aligned Unigene and cDNA sequences to the genome by the BLAT program [114]. We thus identified the sequences of transcripts containing complete genes with introns. After excising introns, the above transcript sequences were used to generate a model for predicting intronless genes using the GeneMark.hmm-P program (http://opal.biology.gatech.edu/GeneMark/). This model was then used in GeneMark.hmm-P to identify the start and stop codons of the genes and to evaluate the gene structures, thereby completing the generation of the test set.

The species specific model used to predict cucumber genes was assessed based on the sensitivity and specificity of gene detection; it was modified (on the basis of alignment analyses performed by the BLAT program) and used for *de novo* prediction of gene structures on all contigs longer than 1000 bp masked for repetitive and TE sequences. To adjust for the specifics of the cucumber genome we modified the default parameters of the model with respect to the minimum and maximum intron lengths and maximum length of intergenic regions. These values were changed, respectively, from 20 nt to 60 nt and from 10,000 to 40,000 nt, for the two last variables.

### Functional annotation

Polypeptides proposed as the result of structural annotation were aligned to the GenBank protein database with the blastp algorithm [105] and the e-value threshold of 1×10^−10^ using the BRAGoMap [115], [116] program. From the ten obtained results, similarities to undescribed or unknown proteins, i.e. putative proteins, unknown proteins, expressed proteins, and hypothetical proteins, were removed. Among the remaining results one protein each with the greatest similarity was left. The identifiers of these proteins were mapped to Uniprot identifiers from the iProClass [117] database and subsequently using the GORetriver [118] program onto the Gene Ontology Consortium [74] classification. A more general GOSlim classification was obtained after using the GOSlimViewer [118] program. The conversion to the GOSlim TAIR classification (http://www.arabidopsis.org) was also performed.

### Comparative analysis of genome sequences of line B10 and 9930

Alignment of the genome sequence contigs of two genetically distant cucumber varieties as well as the analysis of SNP differences between them were performed using the MUMmer 3.22 program for contigs of a minimal length of 1,000 bp. Comparison of the placement of scaffolds of both cucumber lines on chromosomes and visualization of rearrangements were performed using the Mauve ver. 2.3.1 program [119].

### Identification of genes containing ABRE, DRE and ERE elements in their promoters

In order to identify genes which contained in their promoter sequences elements of the ABRE (ACGTGTC), DRE (RCCGAC, where R = A,G) and ERE (AGCCGCC) types, fragments 1,000 bp upstream the start codon (ATG) were taken from databases: http://www.popgenie.org/popgenie1/ - *P. trichocarpa*, ftp://ftp.plantbiology.msu.edu/pub/data/Eukaryotic_Projects/o_sativa/annotation_dbs/ - *O. sativa*. Promoter sequences of the B10 and 9930 lines were isolated from genome sequences on the basis of information on the location of genes assigned to contigs after structural annotation. The *cis* regulatory ABRE, DRE and ERE elements and their combinations were localized in gene promoters of the above species using Perl scripts. Regulatory elements and their combinations in *A. thaliana* gene promoters were sought by the Patmatch program (http://www.arabidopsis.org/cgi-bin/patmatch/nph-patmatch.pl).

### Comparison of protein sequences of *A. thaliana*, *P. trichocarpa*, *O. sativa*, *C. sativus* lines B10 and 9930

Amino acid sequences of gene products of the analyzed species were downloaded from available databases: http://www.popgenie.org/popgenie1/ - *P. trichocarpa*; ftp://ftp.arabidopsis.org/home/tair/Sequences/blast_datasets/TAIR9_blastsets/ – *A. thaliana*, ftp://ftp.plantbiology.msu.edu/pub/data/Eukaryotic_Projects/o_sativa/annotation_dbs/ - *O. sativa*. The cucumber lines B10 and 9930 amino acid sequences of gene products were obtained after the gene prediction. In order to identify groups of orthologous genes, all amino acid sequences of gene products of analyzed species were aligned to each other, we used the BLAST program and the blastp [105] algorithm. Hits with an *e-value* higher than 10^−5^ were rejected. For the prediction of groups of orthologous genes we used OrthoMCL program [120]. Altogether we identified 27,674 groups of orthologous proteins. For further analysis we selected groups which contained at least one gene of each analyzed species or lineages, and complete promoter sequences for all genes. Altogether we described 5,971 groups of orthologous proteins for the *A. thaliana*, *O. sativa*, *P. trichocarpa* and *C. sativus*: lines B10 and 9930 (Dataset S1). Subsequently, on the basis of analysis of promoter sequences of these genes we described common and different groups of orthologous genes in these species and lineages with respect to the occurrence of regulatory elements ABRE, DRE and ERE and combinations thereof.

### Functional classification of genes containing ABRE, DRE and ERE elements in their promoters

The division of *A. thaliana*, *P. trichocarpa*, and *O. sativa* genes into appropriate structural and functional groups was performed on the basis of information obtained from the ftp server: http://www.popgenie.rg/popgenie1/ for analysis of *P. trichocarpa*; ftp://ftp.plantbiology.msu.edu/pub/data/Eukaryotic_Projects/o_sativa/annotation_dbs/ for analysis of *O. sativa*; and ftp://ftp.arabidopsis.org/home/tair/Ontologies/Gene_Ontology/ for *A. thaliana*. The assignment of functional groups for *C. sativus* line B10 proteins was performed by the functional annotation described in the online methods. This division was then unified by manual mapping of numerical identifiers of Generic GO Annotations to the GO Slim TAIR category (www.arabidopsis.org). Genes of the analyzed species were divided, according to function and cellular localization, into appropriate groups: GO Cellular Component, GO Molecular Function and GO Biological Process. In gene promoter sequences for each group we have described the occurrence of *cis* regulatory elements ABRE, DRE, ERE and their combinations ABRE-DRE, ABRE-ERE, DRE-ERE, and ABRE-DRE-ERE in *A. thaliana*, *C. sativus* line B10, *O. sativa* and *P. trichocarpa*.

### Identification of putative transcription factors from *C. sativus* line B10 and 9930

In order to identify putative transcription factors of *C. sativus* line B10 and line 9930, amino acid sequences of gene products were scanned using PFAM [121]. Hits with an *e-value* higher than 10^−5^ were rejected. Hits with the highest similarity to known Arabidopsis AREBs (Abscisic Acid–Responsive Element Binding Protein), ERFs (Ethylene-responsive Element-binding Factor) and DREBs/CBFs (DRE binding proteins) were aligned by ClustalW and phylogenetic trees using Neighbour-Joining method were prepared using MEGA 4 program [122].

### ABA treatment and electrolyte leakage

*Arabidopsis* and cucumber seedlings were subjected to 200 µM Abscisic acid (ABA) (Sigma Aldrich, St. Louis, MO, USA) for 3 days. The last ABA treatment was made 3 hours before freezing treatment. Electrolyte leakage experiments were performed as previously described [123] with the following modifications. Detached *Arabidopsis* and cucumber leaves leaf were placed in test tube and submerged for one hour in −2°C bath containing water and propylene glycol, after which ice crystals were added to initialize crystallization. After one hour of incubation in −2°C samples were cooled to −4°C and kept for additional hour. Samples (at least five replicates for each data point) were thawed overnight in 4°C in 1.5 ml of distilled water. Electrolyte leakage was measured after addition to each sample of 18.5 ml of distilled water. To obtain total electrolyte leakage samples were frozen for at least one hour in −80°C and tested as above.

## Supporting Information

Dataset S1 CRE distributions among two cucumber (*Cucumis sativus*) genotypes.
(XLS)

Dataset S2 Putative transcription factors representing AP2_EREBP and bZIP families from two cucumber (*Cucumis sativus*) genotypes.
(XLS)

Dataset S3 Comparison of the composition of the genes belonging to the different (BP, MF, CC) Gene Ontology functional groups (GOAN) of the two cucumber varieties.
(XLS)

Figure S1 Diagram of cumulative read lengths obtained after unpaired and paired 454 XLR Titanium sequencing of the B10 line. (A) Length of reads after sequencing (blue) UNpaired (UN 8×) and (red) PAired (PA 4×) libraries, before linker trimming. (B) Length of reads after sequencing (purple)UNpaired (UN 8×) library and paired (4×) library, obtained after linker searching and trimming, which resulted in sequences: (red) paired with full linker, (blue) unpaired because of having partial or multi linker and (green) unpaired because of no linker sequences found. 454 XLR Titanium reads were obtained after sequencing of unpaired fragments of 8× genome coverage and sequencing of paired fragments (3 Kbp insert size) with 4× genome coverage. Both types of reads were processed for adapters and linkers trimming with scripts: sff_extract v0.2.3 and sffinfo from Newbler package v2.0.00.20.
(TIF)

Figure S2 Read depth distributions^a^ of the 454 XLR Titanium reads on the cucumber B10 line genome assembly. ^a^The 454 XLR Titanium reads sequencing depth was derived after the Celera assembly step.
(TIF)

Figure S3 Homology of 554 contigs of the 9930 line genome with bacterial genomes. A total of 554 contigs of line 9930 were suspected of representing bacterial genome contigs encoded bacterial proteins. These contigs were aligned to the whole nucleotide GenBank database using the blastn algorithm.
(TIF)

Figure S4 Composition and percentage of bacterial genera represented by suspected contaminating contigs of the 9930 line genome. Cumulative percentage of homologies to the bacterial genera after BLAST alignment of 554 genome contigs of 9930 line suspected of representing bacterial contamination to the GenBank nucleotide database using the blastn algorithm.
(TIF)

Figure S5 Dot-plot of B10 vs. 9930 lines genomes. The Dot-plot figure shows the homology and possible rearrangements between genomic contigs of the two cucumber genotypes. It was made using the MUMmer 3.20 software on repeat-masked contigs longer than 1 Kbp. Red dots represent the same orientation and the blue dots represent the reverse orientation of homolog sequences. Horizontal and vertical axes contain 9930 and B10 genome contigs respectively.
(TIF)

Figure S6 Chromosomal rearrangements between cucumber varieties. Figure shows chromosomal localization of genetic markers and genomic sequences anchored on chromosomes I–VII and a comparison between the B10 and 9930 lines (chromosome numbering is according to the Borszczagowski karyotype [80] (brackets contain Chinese Long [67] karyotype numbering)). a - Chromosome 1 (4), b - Chromosome 2, c – Chromosome 3, d – Chromosome 4 (6), e – Chromosome 5 (1), f – Chromosome 6 (5), g – Chromosome 7. The B10 and 9930 genome scaffolds are shown on the top and middle panels, respectively. The lower panel presents localization of markers on the genetic map. The numbers above top panel represent the relative genomic length of the chromosome. The top lines show chromosomal rearrangements between sequences of two genomes and the bottom lines connect genetic map markers with the sequences of the 9930 line. The 9930 line genotype is set as the reference genome because of the genetic markers originating from this genotype. Boxes of the same color represent scaffolds with high homology between the two cucumber genotypes (or the same scaffolds) and the gradient plot shows the relative homology between them. The B10 genotype boxes above the horizontal line represent scaffold sequences in the same orientation as in the 9930 line, and the boxes below the line indicate scaffold sequences in reverse orientation relative to the 9930 genotype.
(TIF)

Figure S7 Comparison of GOSlim functional groups between two cucumber genomes (B10 and 9930 lines) and *Arabidopsis thaliana*. (A) Biological Process functional groups. (B) Molecular Function functional groups. (C) Cellular Component functional groups.
(TIF)

Figure S8 Distribution of CREs in *C. sativus* line B10 promoters. Promoters from C. sativus were divided into 50 bp fragments and the content of each CRE was determined. Increased density of ABRE, DRE and ERE in sequences less than 300 bp may indicate a major regulatory role of this elements in C. sativus. gene expression.
(TIF)

Figure S9 Structural and functional analysis of *A. thaliana*, *P. trichocarpa*, *O. sativa*, and *C. sativus* genes, whose promoter sequences contain the *cis* regulatory elements ABRE, DRE, ERE and combinations thereof. Analysis was performed on the basis of Gene Ontology annotations (GO). (**A**) Percentage of *A. thaliana*, *P. trichocarpa*, *O. sativa*, *C. sativus* genes which encode proteins of distinct cellular localization. (**B**) Percentage of *A. thaliana*, *P. trichocarpa*, *O. sativa*, *C. sativus* genes which encode proteins of distinct molecular function. (**C**) Percentage of *A. thaliana*, *P. trichocarpa*, *O. sativa*, *C. sativus* genes which encode proteins involved in distinct biological processes.
(TIF)

Figure S10 Structural analysis of promoters of orthologic genes of interest between the two *C. sativus* lines. Sequence alignments were performed using ClustalW software. (**A**) Mutation analysis in promoter sequences. Dotted lines show low homology regions. (**B**) CRE analysis between promoter sequences of the two *C. sativus* lines. The CRE searches were done using the PlantCARE software.
(TIF)

Figure S11 Sequence alignment of selected putative AREBs from *A. thaliana*, *C. sativus* B10 and 9930, *P. trichocarpa* and *O. sativa*. Conserved domains (C1–C3) and bZIP are shown (*top*). Sequences were aligned using ClustalW and edited in Jalview program.
(TIF)

Figure S12 An unrooted phylogenetic tree of selected putative AREBs from *A. thaliana* (dark blue triangle (▴)), *C. sativus* B10 (green circle (•)) and 9930 (khaki circle (•)), *P. trichocarpa* (purple rhombus (♦)) and *O. sativa* (light grey square (▪)). The amino acid sequences from selected putative AREBs were aligned by ClustalW and phylogenetic tree was constructed using MEGA 4.0 and Neighbor-Joining method (bootstrap = 1000).
(TIF)

Figure S13 Sequence alignment of selected putative AP2/ERF-domain containing proteins from *A. thaliana*, *C. sativus* B10 and 9930 and *P. trichocarpa*. Sequences were aligned using ClustalW and edited in Jalview program.
(TIF)

Figure S14 An unrooted phylogenetic tree of selected putative AP2/ERF-domain containing proteins *A. thaliana* (dark blue triangle (▴)), *C. sativus* B10 (green circle (•) and 9930 (khaki circle (•)), *P. trichocarpa* (purple rhombus (♦)) and *O. sativa* (light grey square (▪)). The amino acid sequences from selected putative AP2/ERF-domain containing proteins were aligned by ClustalW and phylogenetic tree was constructed using MEGA 4.0 and Neighbor-Joining method (bootstrap = 1000).
(TIF)

Table S1 Characteristics of BAC end sequences (BESs). Characteristics of BESs obtained from two BAC libraries of B10 line of cucumber. BESs were trimmed and screened for contamination with vector and bacterial genomes using Lucy v1.20 and BLAST. BLAST was also used to find chloroplast and mitochondrion genome sequences.
(XLS)

Table S2 Length characteristics of 454 XLR Titanium unpaired (8×) and paired (4×) reads after trimming of adaptors and linkers. Processing of 454 XLR Titanium 4× paired library fragments, recovered paired reads and also unpaired reads. Fragments from this library which had no linker at all and fragments with partial or multiple linkers were treated as unpaired reads (the same as all of the reads from the 8× unpaired library). The only paired reads were recovered from fragments containing full linker sequences.
(XLS)

Table S3 Results of the B10 line genome assembly steps and comparison with two other cucumber genome assemblies. ^a^N50 refers to the size above which half of the total length of the sequence set can be found.
(XLS)

Table S4 Summary of sequencing reads used by the assembly programs.
(XLS)

Table S5 Characteristics of microsatellite sequences (SSRs) coming from BESs and genome contigs of the B10 line. SSRs (mono- to octanucleotides) longer than 12 nt, with at least 3 repeat unitswere searched in BES and genome contigs of the B10 line using the MISA software. The table shows the minimum of four most frequent motifs for each motif group. The SSR density were calculated as count per Mbp.
(XLS)

Table S6 Characteristics of plant repeat elements found in BESs and genome contigs of the B10 line. Plant repeat elements were classified after BLAST alignment of the B10 line BESs and genome contigs to the database of those elements at Michigan State University.
(XLS)

Table S7 Detailed results of anchoring the sequences from both assembly versions on chromosomes using different methodologies. ^a^The Arachne (version B) assembly was anchored on chromosomes using both the Arachne package specific scripts and BLAST aligning. The Celera (version A) genome sequence was anchored on chromosomes only with the use of BLAST. ^b^Cross-mapping results of Celera vs Arachne genome sequence on chromosomes. The first number represents the Arachne (version B) assembly and the second number represents the Celera (version A) assembly.
(XLS)

Table S8 Results of gene predictions on B10 and 9930 genomes. All gene predictions were done using the GeneMark.hmm ES 3.0 on contigs longer than 1 Kbp. ^a^Contigs assumed to represent bacterial contamination were identified using the BLAST alignments of predicted genes, proteins and contigs containing them in the GenBank submission of 9930 line of Chinese Long variety of cucumber (Huang S., et al., 2009). ^b^As reported in publication by Huang S., et al., 2009. ^c^Mean gene length calculated as mean transcription region length plus half of the mean intergenic region length.
(XLS)

Table S9 Specificity (Sp) and sensitivity (Sn) of GeneMark.hmm ES 3.0 gene prediction model.
(XLS)

Table S10 Basic characterization of functional annotation results for B10 and 9930 genomes. All gene predictions were performed using the GeneMark.hmm ES 3.0 on contigs longer than 1 Kbp. Predicted polypeptides were aligned to the protein GenBank database using BLAST and blastx algorithm. ^a^Contigs assumed to represent bacterial contamination were found using the BLAST alignments of predicted genes, proteins and contigs containing them in the GenBank submission of 9930 line of Chinese Long variety of cucumber (Huang S., et al., 2009). ^b^GeneBank annotated homologies without putative protein, unknown protein, expressed protein, hypothetical protein annotations. ^c^Gene Ontology annotation of proteins was performed by mapping GenBank accession numbers to UniProt identifiers using the iProClass database and then by mapping the identifiers to GOAN using GoRetriver software.
(XLS)

Table S11 SNP and In/Del polymorphism differences between B10 and 9930 line genotypes. SNP polymorphisms were identified using the MUMmer 3.20 software between genomic contigs of B10 line and 9930 line genomes of minimum 1 Kbp length.
(XLS)

Table S12 Table S11_5 - Deteiled characteristics of different length In/Del polimorphism: 1–2 bp (ultra), 3–10 bp (mikro), 11–50 bp (mini), 51–100 bp (midi).
(XLS)

Table S13 Functional differences between cucumber varieties. Table shows characteristics of 38 functional groups of genes, which number significantly differ (minimum 2-fold) between the two cucumber genotypes (B10 and 9930 line). Green and red colors indicate the fold-value of differentiation of genes in functional groups important for Darwinian fitness. BP – Biological Process, MF – Molecular Function, CC – Cellular Component.
(XLS)

Table S14 Statistical analysis of ABREs, DREs and EREs in promoter sequences in *C. sativus* Line B10. ^a^The probability of the element occurring once in N base pairs (N = size of element), based on nucleotide frequency in C. sativus line B10 promoter regions. ^b^Rarity factor is the total number of genes in the C. sativus line B10 genome with this CRE in the proximal 1000 bp promoter, as a fraction of all C. sativus genes. ^c^Enrichment factor is the number of genes with CREs in the proximal 1000 bp promoter divided by the number of genes expected to have this CRE by chance occurrence-based element probability. ^d^average is the number of CREs in promoters with at least one CRE.
(XLS)
