# Supplementary figures and images for: The Genome Sequence of the North-European Cucumber (Cucumis sativus L.) Unravels Evolutionary Adaptation Mechanisms in Plants
Source: PLoS One. 2011 Jul 28;6(7):e22728. doi: 10.1371/journal.pone.0022728 (PMC3145757; doi:10.1371/journal.pone.0022728)

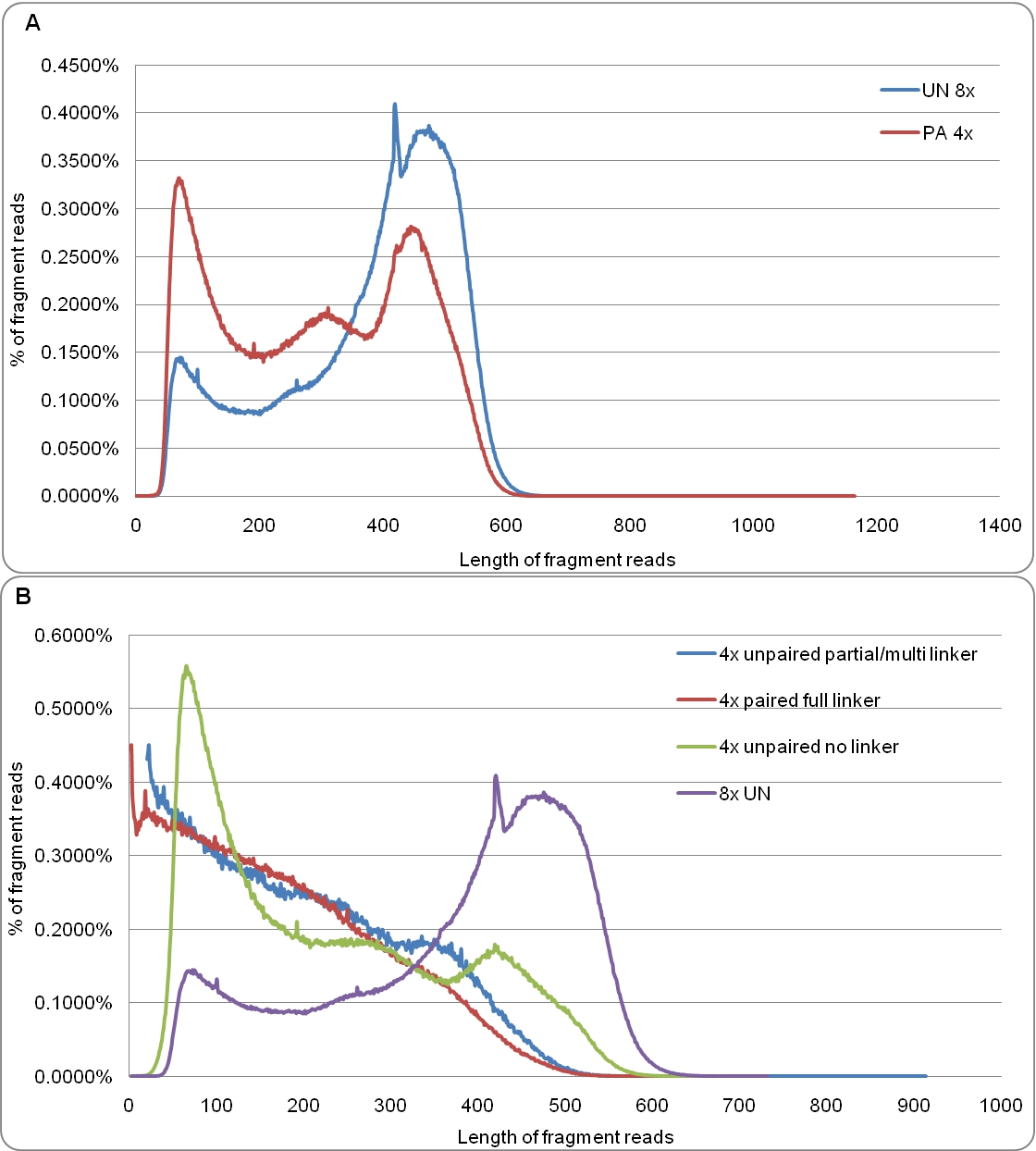

Supplement: Figure S1 — Diagram of cumulative read lengths obtained after unpaired and paired 454 XLR Titanium sequencing of the B10 line. (A) Length of reads after sequencing (blue) UNpaired (UN 8×) and (red) PAired (PA 4×) libraries, before linker trimming. (B) Length of reads after sequencing (purple)UNpaired (UN 8×) library and paired (4×) library, obtained after linker searching and trimming, which resulted in sequences: (red) paired with full linker, (blue) unpaired because of having partial or multi linker and (green) unpaired because of no linker sequences found. 454 XLR Titanium reads were obtained after sequencing of unpaired fragments of 8× genome coverage and sequencing of paired fragments (3 Kbp insert size) with 4× genome coverage. Both types of reads were processed for adapters and linkers trimming with scripts: sff_extract v0.2.3 and sffinfo from Newbler package v2.0.00.20. (TIF) [file pone.0022728.s004.tif]

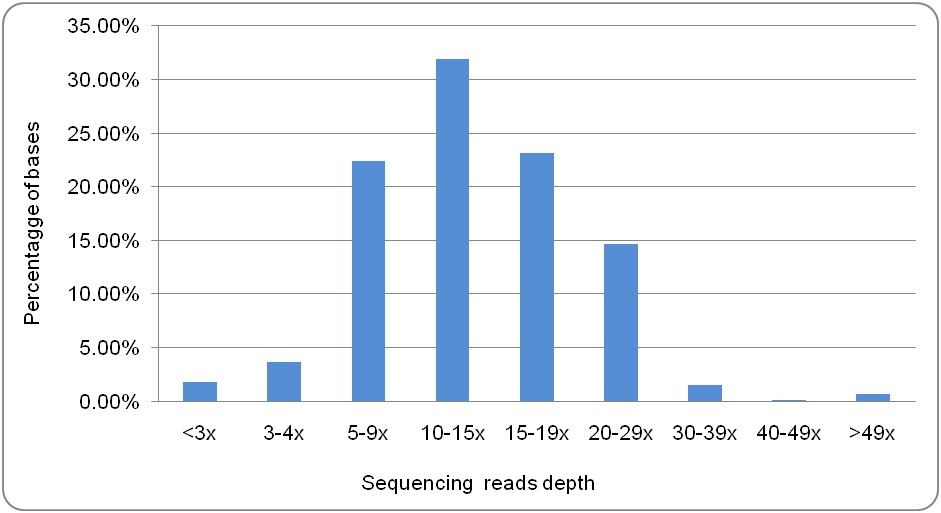

Supplement: Figure S2 — Read depth distributionsa of the 454 XLR Titanium reads on the cucumber B10 line genome assembly. aThe 454 XLR Titanium reads sequencing depth was derived after the Celera assembly step. (TIF) [file pone.0022728.s005.tif]

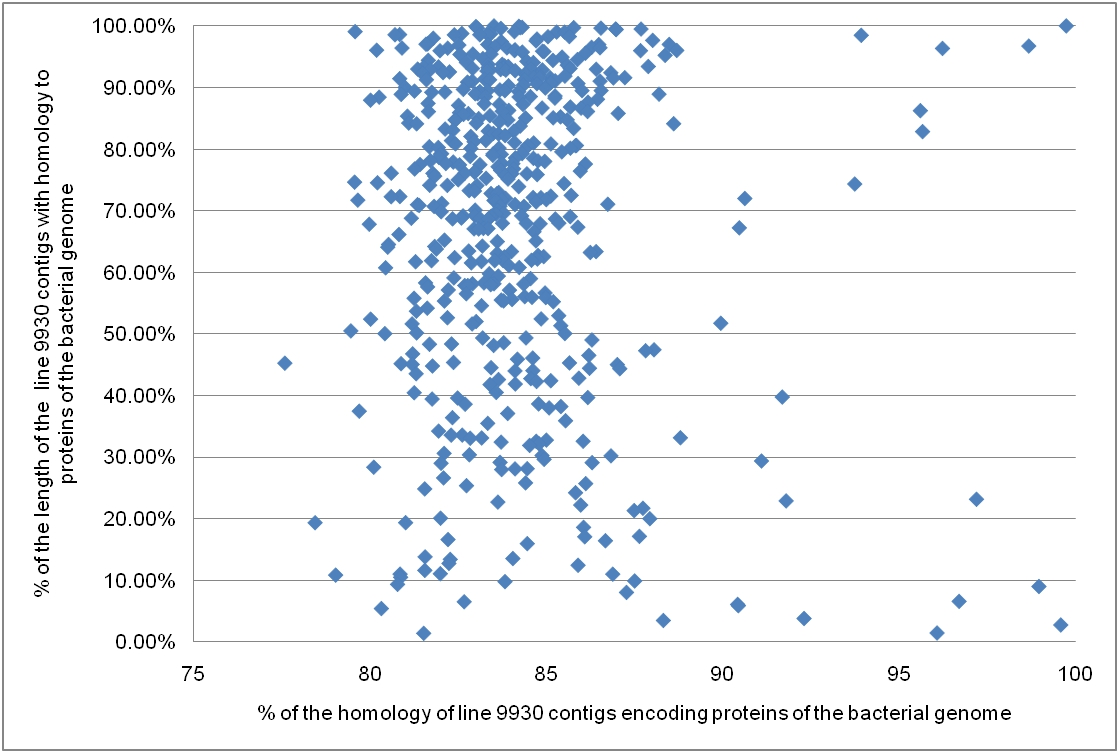

Supplement: Figure S3 — Homology of 554 contigs of the 9930 line genome with bacterial genomes. A total of 554 contigs of line 9930 were suspected of representing bacterial genome contigs encoded bacterial proteins. These contigs were aligned to the whole nucleotide GenBank database using the blastn algorithm. (TIF) [file pone.0022728.s006.tif]

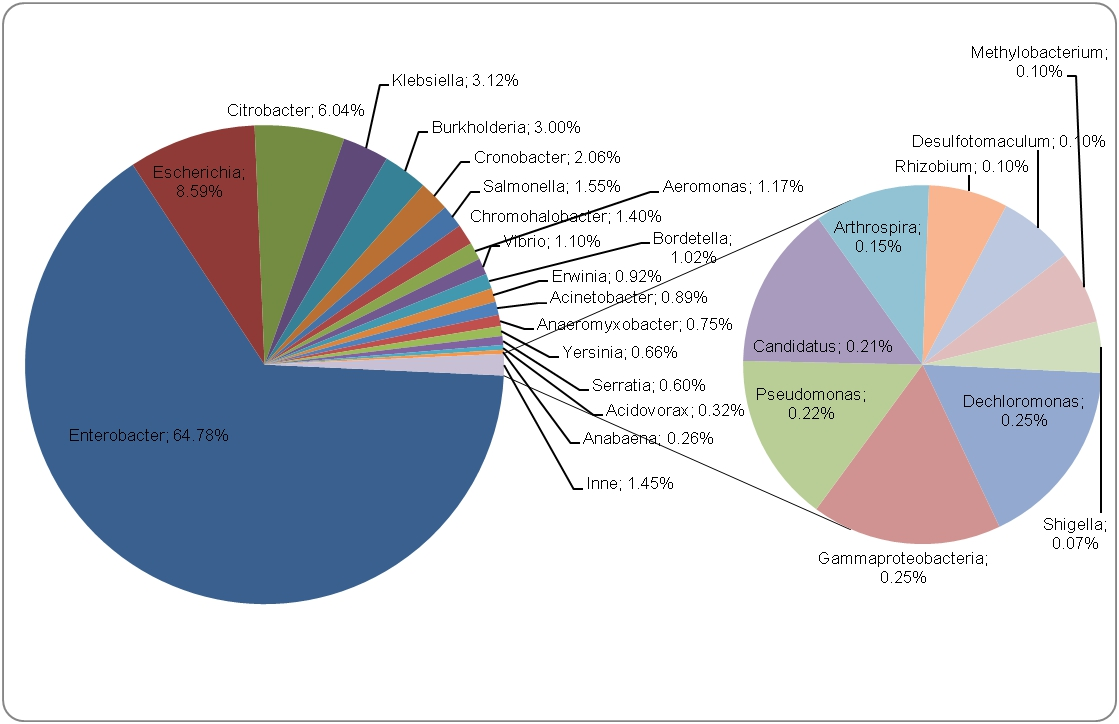

Supplement: Figure S4 — Composition and percentage of bacterial genera represented by suspected contaminating contigs of the 9930 line genome. Cumulative percentage of homologies to the bacterial genera after BLAST alignment of 554 genome contigs of 9930 line suspected of representing bacterial contamination to the GenBank nucleotide database using the blastn algorithm. (TIF) [file pone.0022728.s007.tif]

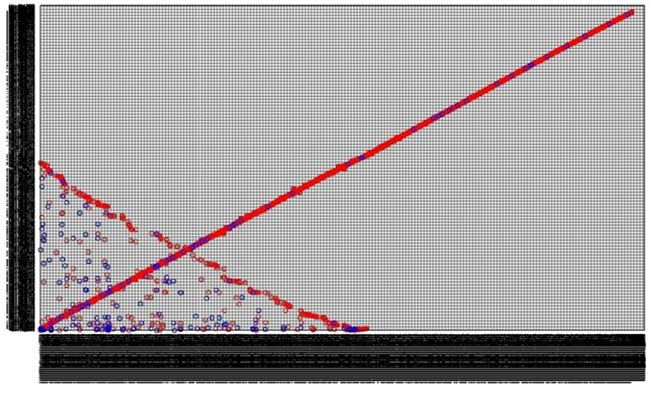

Supplement: Figure S5 — Dot-plot of B10 vs. 9930 lines genomes. The Dot-plot figure shows the homology and possible rearrangements between genomic contigs of the two cucumber genotypes. It was made using the MUMmer 3.20 software on repeat-masked contigs longer than 1 Kbp. Red dots represent the same orientation and the blue dots represent the reverse orientation of homolog sequences. Horizontal and vertical axes contain 9930 and B10 genome contigs respectively. (TIF) [file pone.0022728.s008.tif]

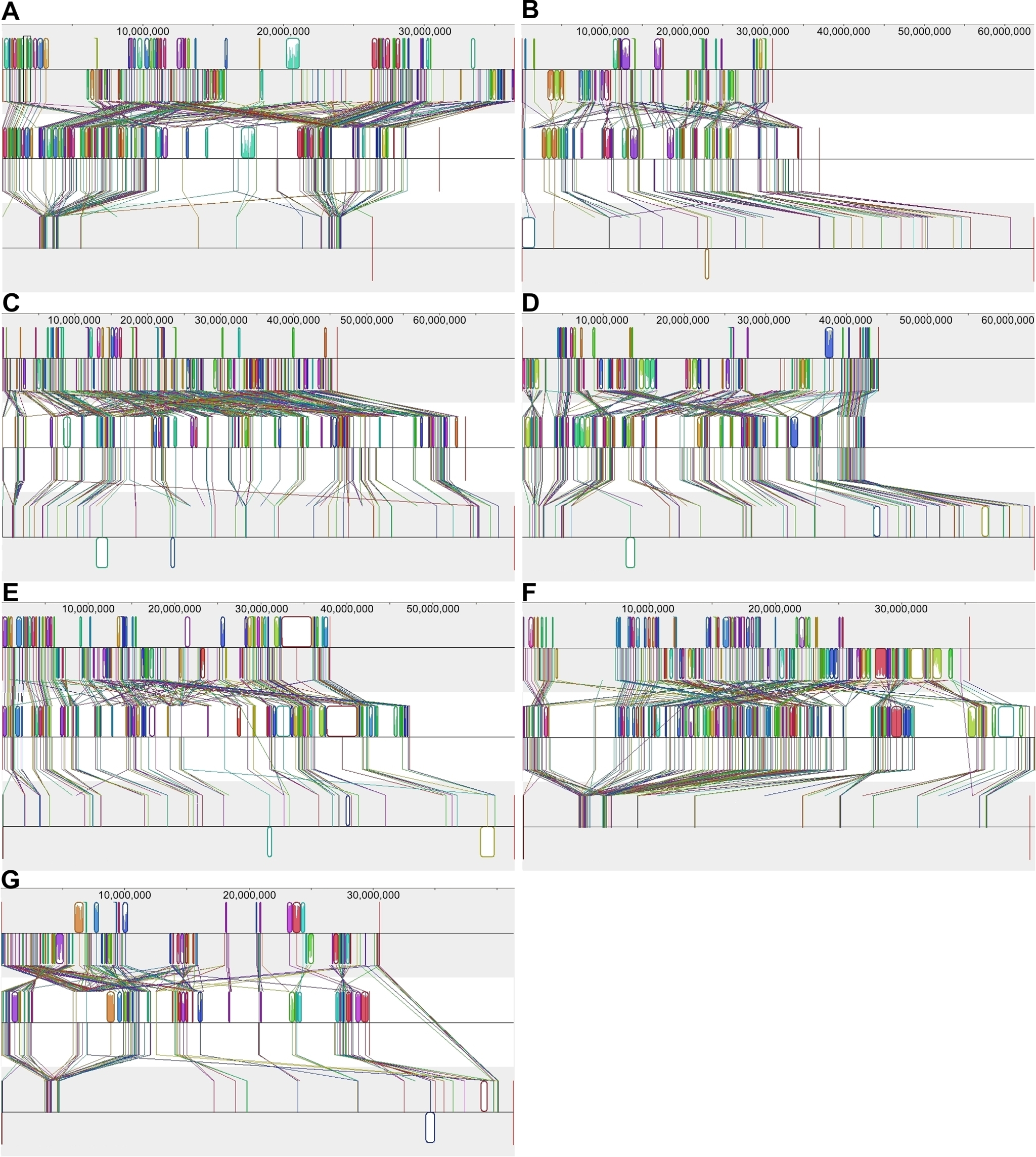

Supplement: Figure S6 — Chromosomal rearrangements between cucumber varieties. Figure shows chromosomal localization of genetic markers and genomic sequences anchored on chromosomes I–VII and a comparison between the B10 and 9930 lines (chromosome numbering is according to the Borszczagowski karyotype [80] (brackets contain Chinese Long [67] karyotype numbering)). a - Chromosome 1 (4), b - Chromosome 2, c – Chromosome 3, d – Chromosome 4 (6), e – Chromosome 5 (1), f – Chromosome 6 (5), g – Chromosome 7. The B10 and 9930 genome scaffolds are shown on the top and middle panels, respectively. The lower panel presents localization of markers on the genetic map. The numbers above top panel represent the relative genomic length of the chromosome. The top lines show chromosomal rearrangements between sequences of two genomes and the bottom lines connect genetic map markers with the sequences of the 9930 line. The 9930 line genotype is set as the reference genome because of the genetic markers originating from this genotype. Boxes of the same color represent scaffolds with high homology between the two cucumber genotypes (or the same scaffolds) and the gradient plot shows the relative homology between them. The B10 genotype boxes above the horizontal line represent scaffold sequences in the same orientation as in the 9930 line, and the boxes below the line indicate scaffold sequences in reverse orientation relative to the 9930 genotype. (TIF) [file pone.0022728.s009.tif]

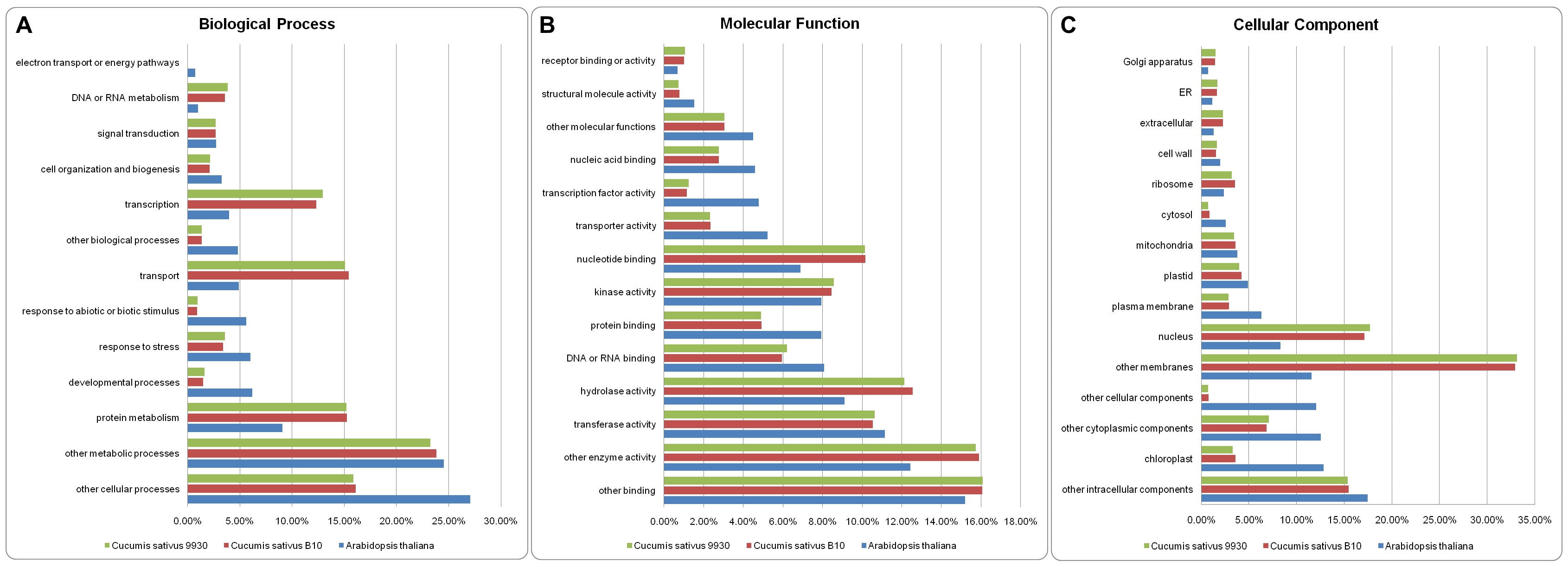

Supplement: Figure S7 — Comparison of GOSlim functional groups between two cucumber genomes (B10 and 9930 lines) and Arabidopsis thaliana. (A) Biological Process functional groups. (B) Molecular Function functional groups. (C) Cellular Component functional groups. (TIF) [file pone.0022728.s010.tif]

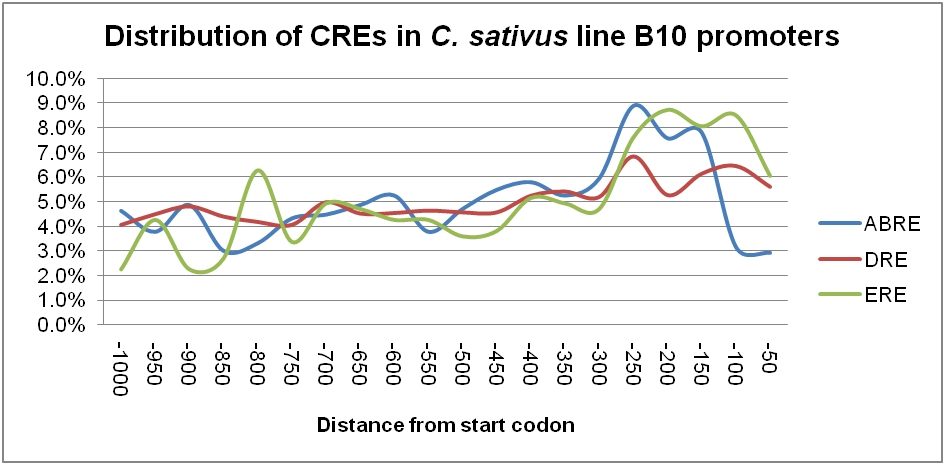

Supplement: Figure S8 — Distribution of CREs in C. sativus line B10 promoters. Promoters from C. sativus were divided into 50 bp fragments and the content of each CRE was determined. Increased density of ABRE, DRE and ERE in sequences less than 300 bp may indicate a major regulatory role of this elements in C. sativus. gene expression. (TIF) [file pone.0022728.s011.tif]

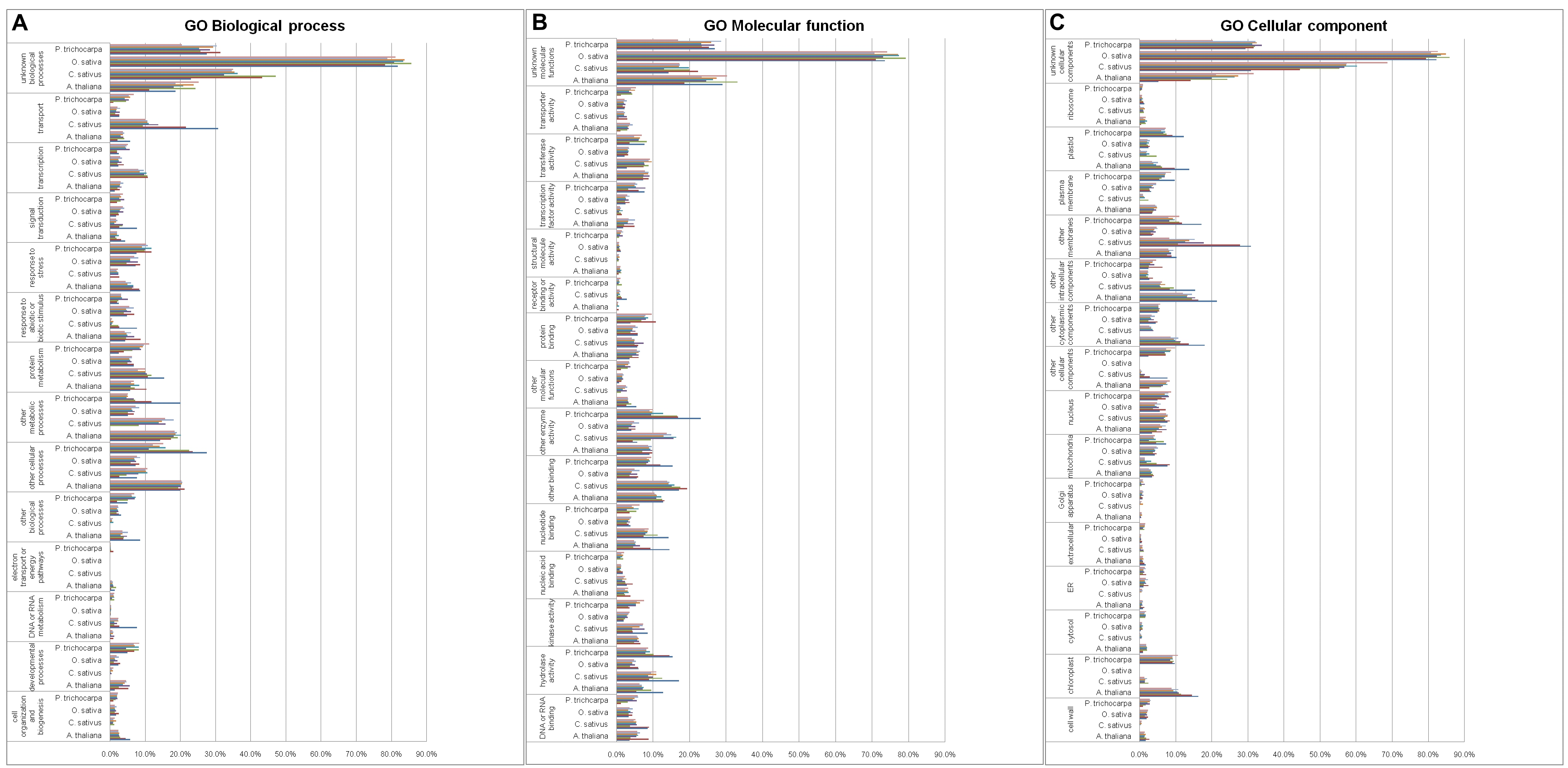

Supplement: Figure S9 — Structural and functional analysis of A. thaliana, P. trichocarpa, O. sativa, and C. sativus genes, whose promoter sequences contain the cis regulatory elements ABRE, DRE, ERE and combinations thereof. Analysis was performed on the basis of Gene Ontology annotations (GO). (A) Percentage of A. thaliana, P. trichocarpa, O. sativa, C. sativus genes which encode proteins of distinct cellular localization. (B) Percentage of A. thaliana, P. trichocarpa, O. sativa, C. sativus genes which encode proteins of distinct molecular function. (C) Percentage of A. thaliana, P. trichocarpa, O. sativa, C. sativus genes which encode proteins involved in distinct biological processes. (TIF) [file pone.0022728.s012.tif]

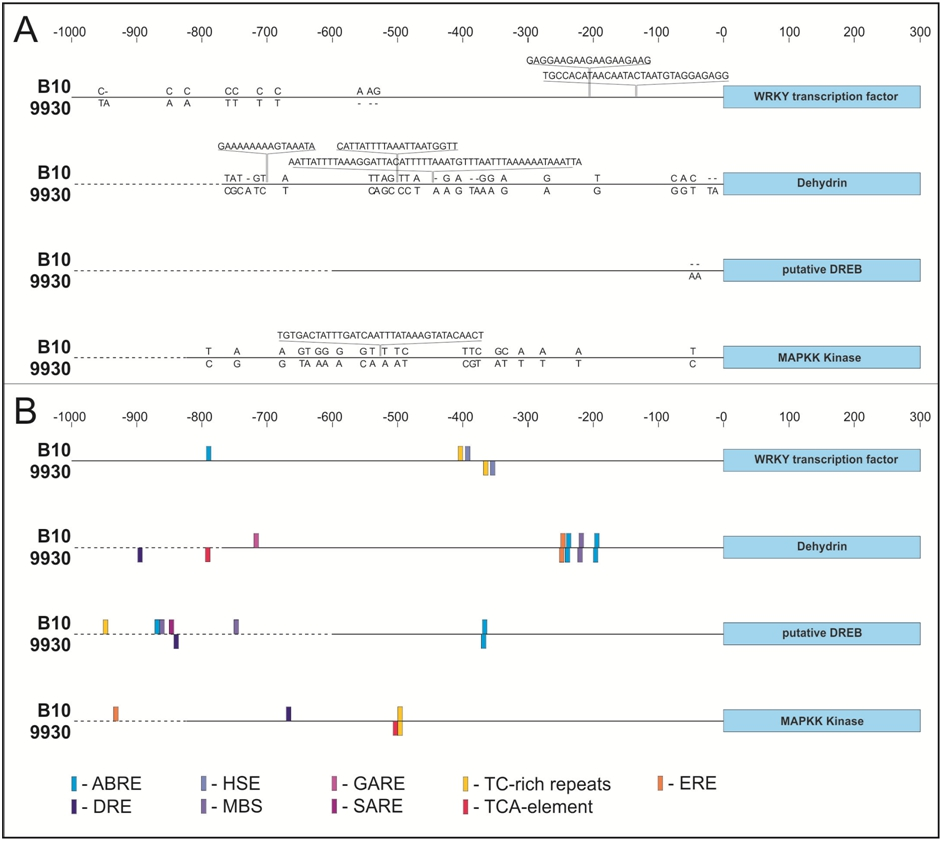

Supplement: Figure S10 — Structural analysis of promoters of orthologic genes of interest between the two C. sativus lines. Sequence alignments were performed using ClustalW software. (A) Mutation analysis in promoter sequences. Dotted lines show low homology regions. (B) CRE analysis between promoter sequences of the two C. sativus lines. The CRE searches were done using the PlantCARE software. (TIF) [file pone.0022728.s013.tif]

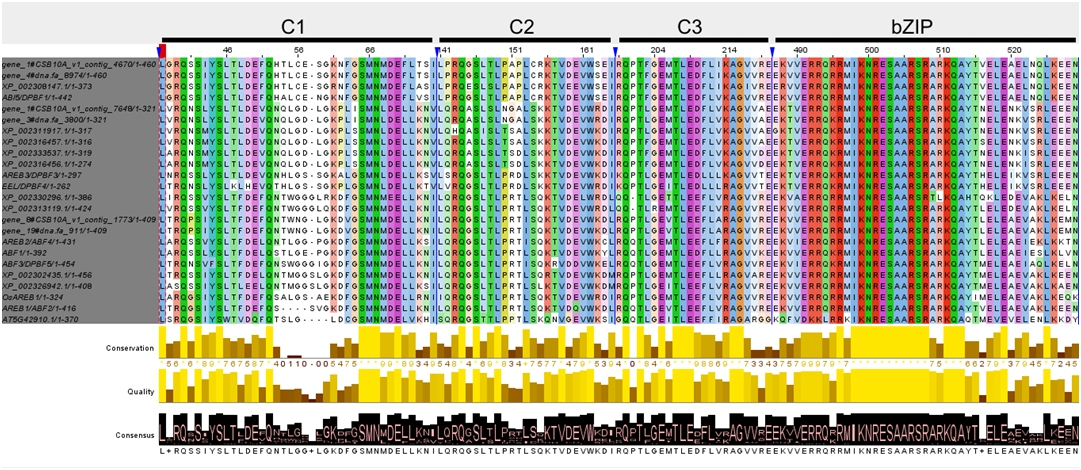

Supplement: Figure S11 — Sequence alignment of selected putative AREBs from A. thaliana, C. sativus B10 and 9930, P. trichocarpa and O. sativa. Conserved domains (C1–C3) and bZIP are shown (top). Sequences were aligned using ClustalW and edited in Jalview program. (TIF) [file pone.0022728.s014.tif]

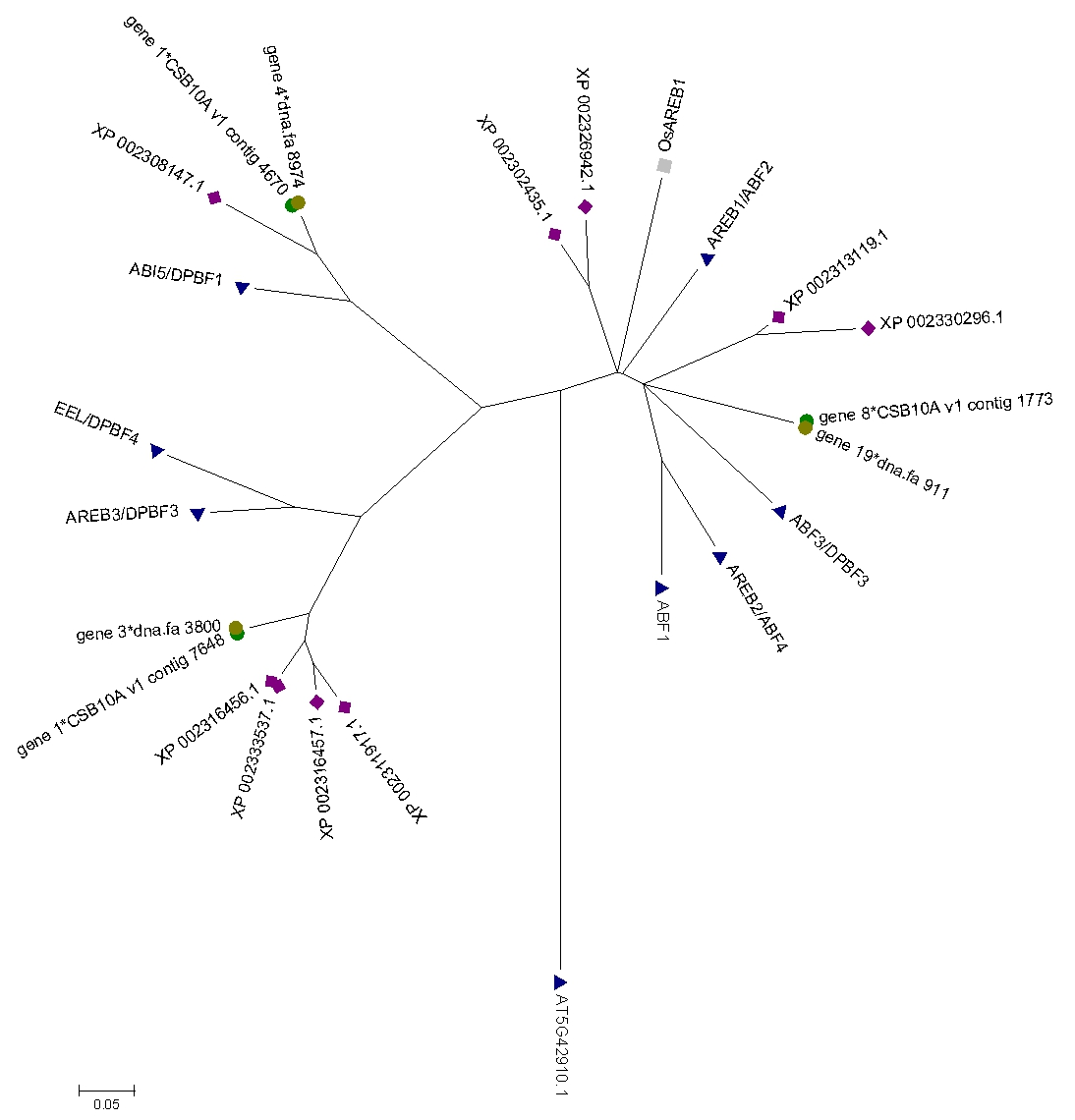

Supplement: Figure S12 — An unrooted phylogenetic tree of selected putative AREBs from A. thaliana (dark blue triangle (▴)), C. sativus B10 (green circle (•)) and 9930 (khaki circle (•)), P. trichocarpa (purple rhombus (♦)) and O. sativa (light grey square (▪)). The amino acid sequences from selected putative AREBs were aligned by ClustalW and phylogenetic tree was constructed using MEGA 4.0 and Neighbor-Joining method (bootstrap = 1000). (TIF) [file pone.0022728.s015.tif]

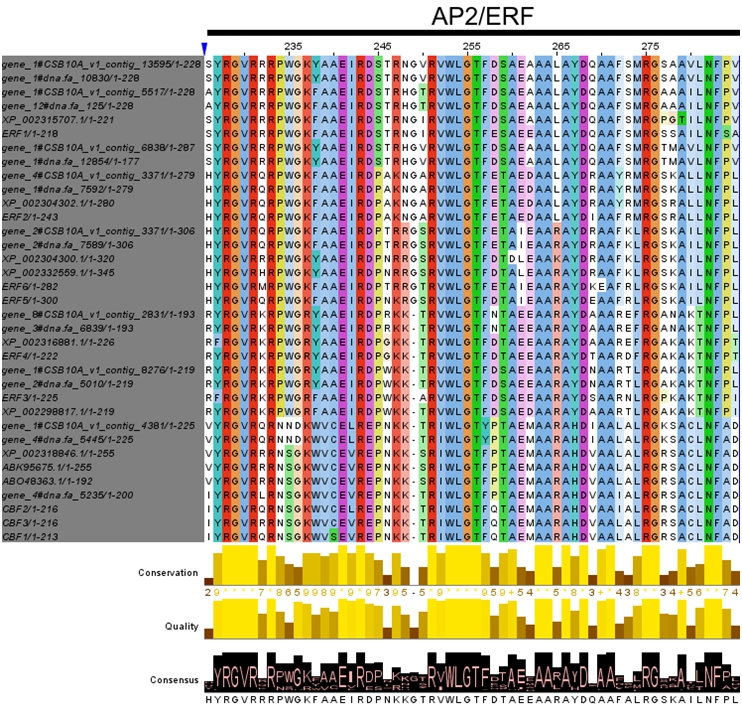

Supplement: Figure S13 — Sequence alignment of selected putative AP2/ERF-domain containing proteins from A. thaliana, C. sativus B10 and 9930 and P. trichocarpa. Sequences were aligned using ClustalW and edited in Jalview program. (TIF) [file pone.0022728.s016.tif]

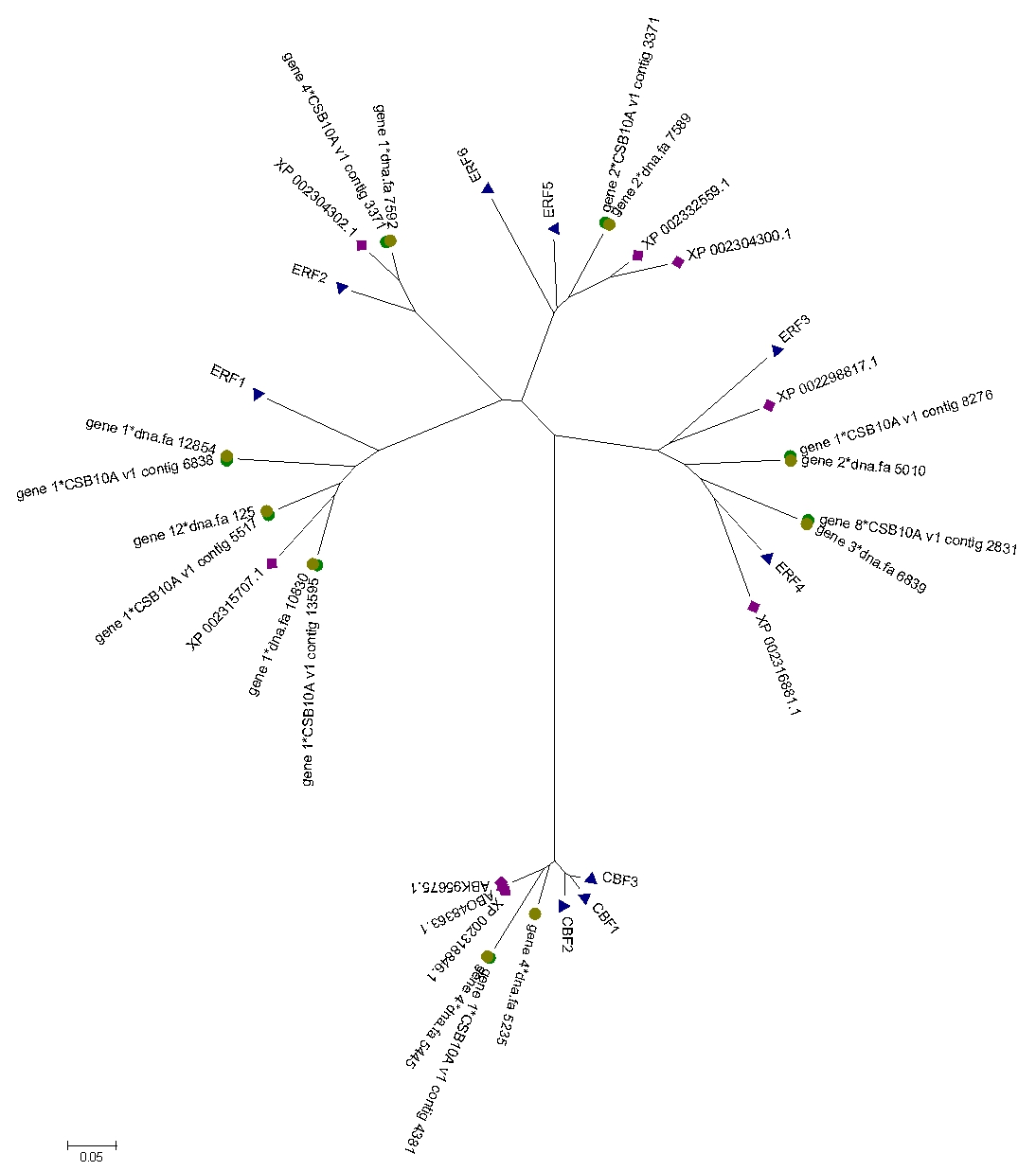

Supplement: Figure S14 — An unrooted phylogenetic tree of selected putative AP2/ERF-domain containing proteins A. thaliana (dark blue triangle (▴)), C. sativus B10 (green circle (•) and 9930 (khaki circle (•)), P. trichocarpa (purple rhombus (♦)) and O. sativa (light grey square (▪)). The amino acid sequences from selected putative AP2/ERF-domain containing proteins were aligned by ClustalW and phylogenetic tree was constructed using MEGA 4.0 and Neighbor-Joining method (bootstrap = 1000). (TIF) [file pone.0022728.s017.tif]
